# Identification of Δ-1-pyrroline-5-carboxylate derived biomarkers for hyperprolinemia type II

**DOI:** 10.1038/s42003-022-03960-2

**Published:** 2022-09-21

**Authors:** Jona Merx, Rianne E. van Outersterp, Udo F. H. Engelke, Veronique Hendriks, Ron A. Wevers, Marleen C. D. G. Huigen, Huub W. A. H. Waterval, Irene M. L. W. Körver-Keularts, Jasmin Mecinović, Floris P. J. T. Rutjes, Jos Oomens, Karlien L. M. Coene, Jonathan Martens, Thomas J. Boltje

**Affiliations:** 1 Radboud University, Institute for Molecules and Materials, Synthetic Organic Chemistry, Heyendaalseweg 135, 6525 AJ Nijmegen, The Netherlands; 2 Radboud University, Institute for Molecules and Materials, FELIX Laboratory, Toernooiveld 7, 6525 ED Nijmegen, the Netherlands; 3 Department of Laboratory Medicine, Translational Metabolic Laboratory, Radboud University Medical Center, Geert Grooteplein Zuid 10, 6525 GA Nijmegen, the Netherlands; 4 United for Metabolic Disease, UMD, Amsterdam, The Netherlands; 5 Department of Clinical Genetics, Maastricht University Medical Center, Maastricht, The Netherlands; 6 Department of Physics, Chemistry and Pharmacy, University of Southern Denmark, Campusvej 55, 5230 Odense, Denmark; 7 Department of Clinical Chemistry and Hematology, Elisabeth-TweeSteden Hospital, Tilburg, The Netherlands

**Keywords:** Metabolomics, Diagnostic markers

## Abstract

Hyperprolinemia type II (HPII) is an inborn error of metabolism due to genetic variants in *ALDH4A1*, leading to a deficiency in Δ-1-pyrroline-5-carboxylate (P5C) dehydrogenase. This leads to an accumulation of toxic levels of P5C, an intermediate in proline catabolism. The accumulating P5C spontaneously reacts with, and inactivates, pyridoxal 5’-phosphate, a crucial cofactor for many enzymatic processes, which is thought to be the pathophysiological mechanism for HPII. Here, we describe the use of a combination of LC-QTOF untargeted metabolomics, NMR spectroscopy and infrared ion spectroscopy (IRIS) to identify and characterize biomarkers for HPII that result of the spontaneous reaction of P5C with malonic acid and acetoacetic acid. We show that these biomarkers can differentiate between HPI, caused by a deficiency of proline oxidase activity, and HPII. The elucidation of their molecular structures yields insights into the disease pathophysiology of HPII.

## Abstract (web summary)

Combined metabolomics, NMR, and, IRIS identify biomarkers of hyperprolinemia type II (HPII) distinct from HPI and similar metabolic signatures as in patients with pyridoxine-dependent epilepsy.

## Introduction

**Fig. 1:**
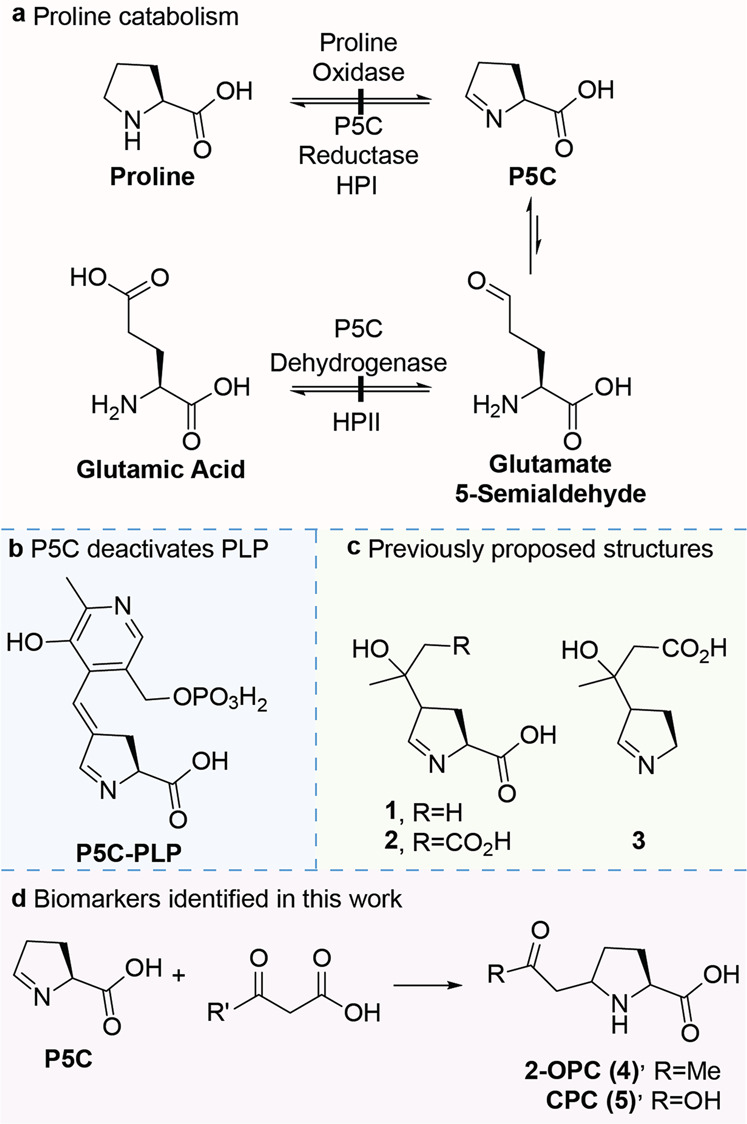
The deficient metabolic conversion of P5C to glutamic acid leads to aberrant reactivity. **a** Deficiency of P5C dehydrogenase leads to the accumulation of P5C and glutamate 5-semialdehyde. **b** Reaction of P5C with PLP leads to the deactivation of the latter. **c** Previously proposed biomarkers for HPII^6^. **d** Biomarkers of P5C with 1,3-dicarbonyl compounds were identified in this study.

Hyperprolinemia type II (HPII, OMIM 239510) is a rare inborn error of metabolism (IEM) for which the main biochemical hallmark is the accumulation of proline in body fluids. HPII is caused by a deficiency of the *ALDH4A1*-encoded Δ-1-pyrroline-5-carboxylate dehydrogenase, which is the enzyme responsible for the second step in the degradation of proline. This step involves the conversion of l-glutamate 5-semialdehyde, which is in equilibrium with its cyclic counterpart Δ-1-pyrroline-5-carboxylate (P5C), to glutamic acid (Fig. 1a)^1,2^. Clinically, HPII is characterized by convulsions in the childhood period, which can be provoked by a concurrent infection^3^. It was proposed that a secondary vitamin B6 deficiency contributes to the convulsions, as accumulating P5C inactivates the biologically active form of vitamin B6, pyridoxal phosphate (PLP), by a spontaneous reaction to P5C-PLP (Fig. 1b)^4,5^. Furthermore, P5C was found to react with acetoacetic acid and it was hypothesized, based on an analogy with the reaction of P5C with PLP, that this leads to the formation of adducts **1**-**3** (Fig. 1c)^6^.

By analogy, PLP inactivation is thought to be the main pathophysiological mechanism in pyridoxine-dependent epilepsy (PDE-ALDH7A1; OMIM 266100), an IEM in the metabolism of lysine, resulting in early onset refractory seizures, which are treatable by the administration of pyridoxine. In this inborn error of lysine catabolism, the cyclic imine Δ-1-pyrroline-6-carboxylate (P6C) accumulates and reacts through the nucleophilic enamine tautomer with PLP, leading to a PLP deficiency^7^. Through an untargeted metabolomics approach (Next Generation Metabolic Screening, NGMS)^8^, our team has recently identified biomarkers for PDE-ALDH7A1. These biomarkers are the result of P6C reacting with acetoacetic acid or malonic acid^9,10^. Due to the structural similarity between the cyclic imines P6C and P5C, we hypothesized that this mechanism of adduct formation would also occur for the reaction of P5C, leading to the formation of (2 *R*)-5-(2-oxopropyl)pyrrolidine-2-carboxylic acid (2-OPC, **4**) and (2 *R*)-5-(carboxymethyl)pyrrolidine-2-carboxylic acid (CPC, **5**) (Fig. 1d) and that the previously proposed structures of the reaction product of P5C with acetoacetic acid, biomarkers **1-2** (Fig. 1c), were likely incorrectly assigned in previous studies^6^.

Using our previously established approach, combining untargeted metabolomics with infrared ion spectroscopy (IRIS) and synthesis of reference standards, we show here that P5C reacts with acetoacetic acid or malonic acid in an analogous manner as we previously elucidated for P6C^9^, leading to the formation of biomarkers 2-OPC (**4**) and CPC (**5**), respectively (Fig. 1d). These findings further confirm a general biochemical reaction between cyclic imino acids and 1,3-dicarbonyl compounds. Moreover, the identified metabolites have the potential for use as biomarkers for the diagnosis of HPII and give insights into the pathophysiology of this disease.

## Results

### LCMS analysis of HPII samples

Targeted metabolomics of plasma samples of three HPII patients from two independent families revealed significantly increased features compared to controls. Besides the known biomarkers for HPII, proline, 2-pyrroloylglycine, and pyrrole-2-carboxylic acid^11–13^, three additional features were identified (Table 1). More specifically, two features, A and B, both with a mass to charge ratio (*m/z*) of 170.0823 (retention time 1.16 and 1.19 min, respectively), were significantly increased in fluids of HPII patients compared to controls; these biomarkers are also observed in urine and plasma of patients in ketosis (Figure S1). As well, feature C, with *m/z* 172.0615 (retention time 0.82 min, respectively), was found to be increased in all HPII patient samples, albeit to a lesser degree. We note that none of these features were found to increase in concentration in the plasma sample of an HPI patient (SI Figs. S1, S2 and S3) and enable the differentiation of HPI and HPII. Annotation of the known features, proline, hydroxyproline, 2-pyrroloylglycine, and pyrrole-2-carboxylic acid, based on the detection of their *m/z* in LCMS (SI Figs. S4–S7), was performed based on the Human Metabolome Database (HMDB) following a bioinformatic protocol previously described by Coene et al.^10^.

**Table 1:** Identified features by untargeted metabolomics in HPII body fluids.

Feature	m/z	RT (min)	Plasma
A [M-H]^-^	170.0823	1.16	↑
B [M-H]^-^	170.0615	1.19	↑
C [M-H]^-^	172.0615	0.82	↑
^13^C-Proline [M + H]^+^	117.0739	0.70	↑
Hydroxyproline [M + H]^+^	130.0499	1.70	↑
2-Pyrroloyl-glycine [M-H]^-^	167.0456	3.72	↑
Pyrrole-2-carboxylic acid [M-H]^-^	110.0248	4.57	↑

**Fig. 2:**
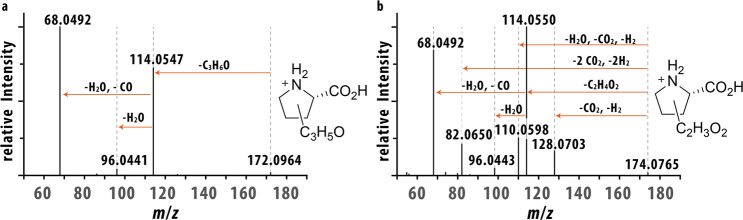
Collision-induced dissociation (CID) MS/MS spectra of features A and C isolated from HPII plasma samples. **a** CID MS/MS spectra of feature A (*m/z* 172.0964). **b** CID MS/MS spectra of feature C (*m/z* 174.0765).

Characterization of the other detected features was first attempted using collision-induced dissociation (CID) MS/MS in positive mode. For features A and B, the primary fragment corresponded to neutral loss of C_3_H_6_O (Δm = 2.6 ppm), resulting in a fragment with *m/z* 114.0547, which further fragmented to *m/z* 96.044 and *m/z* 68.049 after neutral losses of H_2_O (Δm = 3.1 ppm) and CO + H_2_O (Δm = 4.4 ppm), respectively (Fig. 2a). A similar fragmentation of feature C to *m/z* 114.0550 was found, indicating a neutral loss of C_2_H_4_O_2_ (Δm = 3.5 ppm), with secondary fragmentation to both *m/z* 96.0443 (Δm = 1.0 ppm) and *m/z* 68.0495 (Δm = 4.4 ppm). Unique for biomarker C, is the fragmentation to *m/z* 128.0703, 110.0598, and 82.0650, corresponding to a neutral loss of CO_2_ + H_2_ (Δm = 2.3), CO_2_ + H_2_O + H_2_ (Δm = 1.8), and 2CO_2_ + 2H_2_ (Δm = 1.2), respectively (Fig. 2b). Of particular interest is the *m/z* 114.055 fragment in both spectra which corresponds to the mass of [P5C + H]^+^ (Δm = 3.5 ppm), the accumulating metabolite of HPII.

### Identification of features A and B using quantum-chemical calculations

A similar fragmentation pattern was observed for the P6C analogs we previously annotated^9,10^, namely the neutral loss of C_3_H_6_O for feature A and B and C_2_H_4_O_2_ for feature C. We, therefore, hypothesized a similar structure and connectivity of these fragments to the P5C-core leading to the proposed structures of 2-OPC (**4**) and CPC (**5**) (Fig. 1d).

**Fig. 3:**
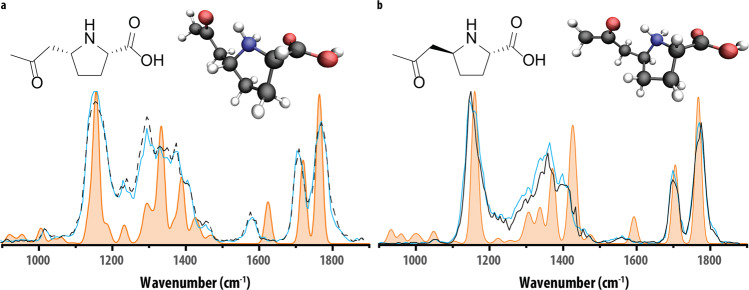
Confirmation of the structural assignment of feature A and B with synthetic reference standards. Comparison of the experimental IR spectra of **a** feature A (black trace) and reference standard **4*cis*** (blue line) with theoretical IR spectrum of **4**_***cis***_ (orange trace), **b** feature A (black trace) and reference standard **4**_***trans***_ (blue line) with theoretical IR spectrum of **4**_***trans***_ (orange trace). Molecular structures corresponding to the protonated reference standards are inlayed in each panel.

In an effort to identify features A and B, they were further characterized using IRIS. This technique enables the recording of IR spectra of mass-isolated ions inside a mass spectrometer, providing sensitive information on the exact molecular structure of the ion. Initial structural elucidation is possible via comparison to quantum-chemically predicted IR spectra of candidate structures. IR spectra were recorded of the mass-isolated protonated ions (*m/z* 172.0968) of feature A and B, isolated from HPII urine samples using a hydrophilic interaction liquid chromatography (HILIC) method, which resulted in baseline separation of the features, which could not be accomplished by the reversed-phase method used for the untargeted screening. Figure 3 shows the comparison of the experimental spectra of features A and B (black traces) to the theoretical reference spectra (orange-filled traces), revealing a qualitative similarity indicative of the general chemical structure of feature A and B.

In particular, the C=O stretching vibrations belonging to the carbonyl-groups calculated for structure **4**, at ~1710 and ~1750 cm^−1^, show a good overlap with the experimentally obtained spectra for both feature A and B. For both features, an excellent overlap is found for the intense C-OH stretch of the carboxylic acid, predicted at 1160 cm^−1^. In contrast, the calculated frequency belonging to the NH_2_-vibration does not match well with the experimentally obtained spectra for features A and B. This discrepancy can be attributed to the anharmonic behavior of the NH bending mode, resulting in a redshift in spectra predicted within the harmonic approximation^14,15^, as was noted before for the corresponding P6C biomarker^9^. Overall the spectral region between 1200 and 1500 cm^−1^ is adequately predicted by the computed spectra. The peak-shape in this range, distinguishing the two features due to the difference in stereochemistry, is reproduced well in the calculated spectra. Based on this difference in peak-shape, we tentatively assign feature A as **4**_***cis***_ and B as **4**_***trans***_

### Synthesis of reference standards for A-C

**Fig. 4:**
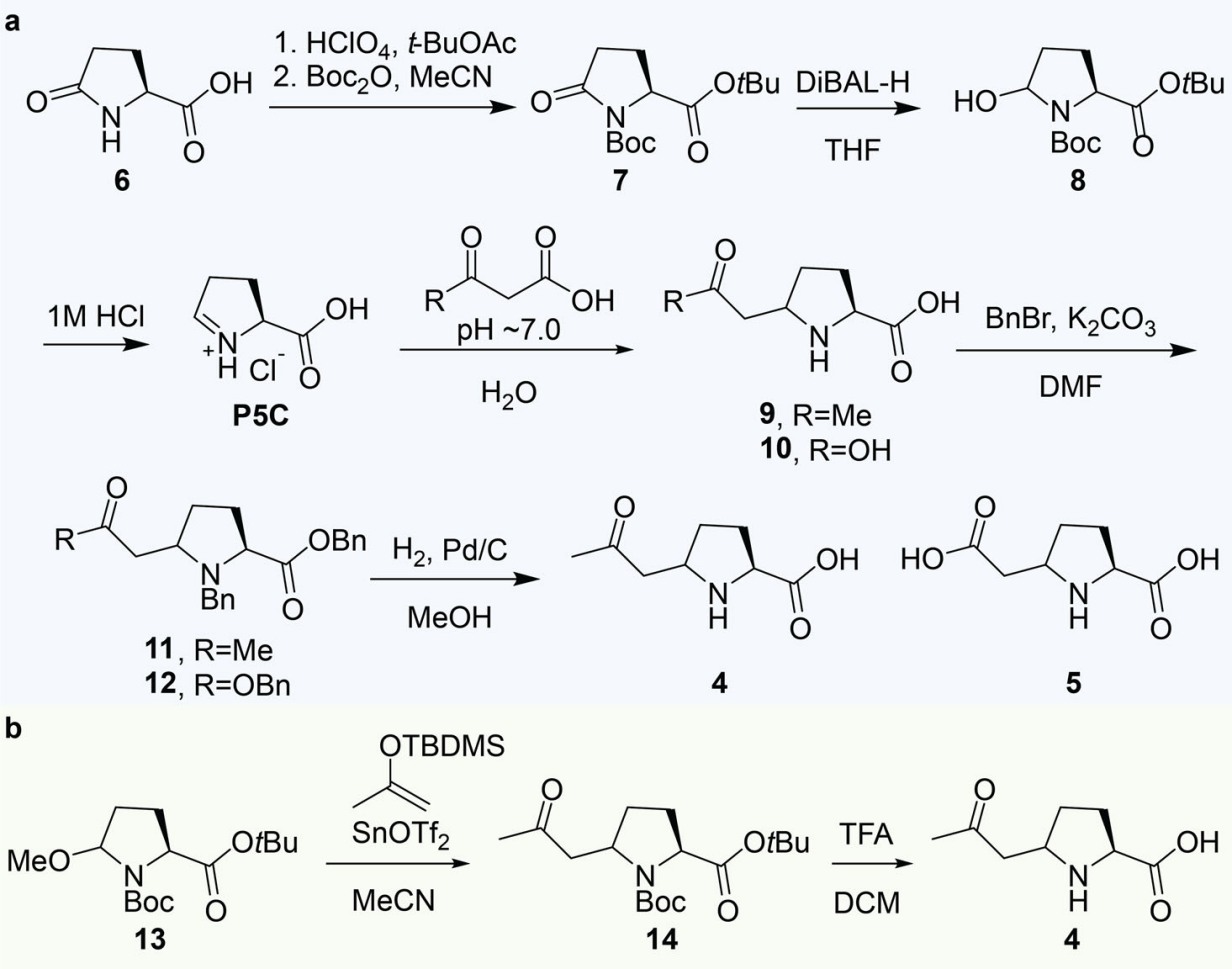
Synthesis of the standards used for the confirmation of the biomarkers. Biomarkers **4-5** were synthesized via (**a**) an incubation of malonic acid or acetoacetic acid with P5C and **b** the mixture of diasteroeisomers of **4** was synthesized via  N-acyliminium ion chemistry.

To confirm the identity of feature A and B, as well as to prove that the structures proposed can arise from the reaction of P5C with 1,3-dicarbonyl compounds, the reference standards were synthesized. The cyclic imine P5C was incubated with the likely reaction partners, acetoacetic acid and malonic acid, in vitro under physiologically relevant conditions. P5C was prepared by the acidic deprotection of **8**, obtained from the reduction of lactam **7** with di-*iso*butylaluminium hydride (DIBAL-H) (Fig. 4a). Incubation of P5C with acetoacetic acid or malonic acid at neutral pH in aqueous media led to the formation of a mixture containing the mass over charge ratio observed for biomarker A-B (**9**) and C (**10**), respectively. For purification purposes, the mixture was benzylated, resulting in the isolation of **11** and **12**, respectively. Subsequent hydrogenation allowed the isolation of both **4** and **5**, although only the *cis* stereoisomer of **4** could be isolated with acceptable purity (Fig. 4a). To synthesize a mixture of diastereoisomers, we turned to *N*-acyliminium ion chemistry. The addition of the methyl ketone fragment was achieved by Lewis acid activation of the *N*,*O* acetal **13** in the presence of the *tert*-butyldimethylsilyl (TBDMS) protected enol ether of acetone. The resulting inseparable diastereoisomers were deprotected with TFA to yield **4** as a mixture of diastereoisomers (Fig. 4b).

### Confirmation of feature A and B using IR spectra of synthetically obtained reference standards

IR spectra were recorded for the protonated ions of both (HILIC-separated) diastereoisomers of the synthetic reference compound **4**. A comparison of the IR spectra (Fig. 3, blue traces), with the measured features A and B (black traces), yielded an excellent spectral match. The minor deviations can be attributed to fluctuations in the experimental conditions between measurements, such as the laser power. The combination of NMR characterization of the synthesized standards and excellent spectral match in the IRIS experiment of the synthesized standards with the metabolites confirms the identity of features A and B. Comparison of the retention time and IRIS spectra of the biomarkers in patients with the single isolated diastereoisomer, **4**_***cis***_, allowed us to definitively assign the stereochemistry of biomarker A and B as **4**_***cis***_ and **4**_***trans***_ respectively (SI Figs. S8 and S9).

### Confirmation of biomarker C

Biomarker C has a lower abundance in the patient samples and, moreover, is poorly retained by the standard RPLC method used for untargeted screening^16^. Additionally, the sensitivity of the HILIC method used to separate biomarker A and B was insufficient to detect biomarker C. This prevented the use of IRIS for the identification of biomarker C, and we, therefore, rely on LC and MS/MS to elucidate the structure of feature C. CID of both the reference standard as well as the mass-selected ions of feature C resulted in the same fragmentation pattern (SI Fig. S10). Taken together with the IR-based assignments of metabolites A and B and the analogous reaction product observed for PDE^9,10^, we assign feature C to be the reaction product of P5C with malonic acid as a mixture of inseparable diastereoisomers.

## Discussion

**Fig. 5:**
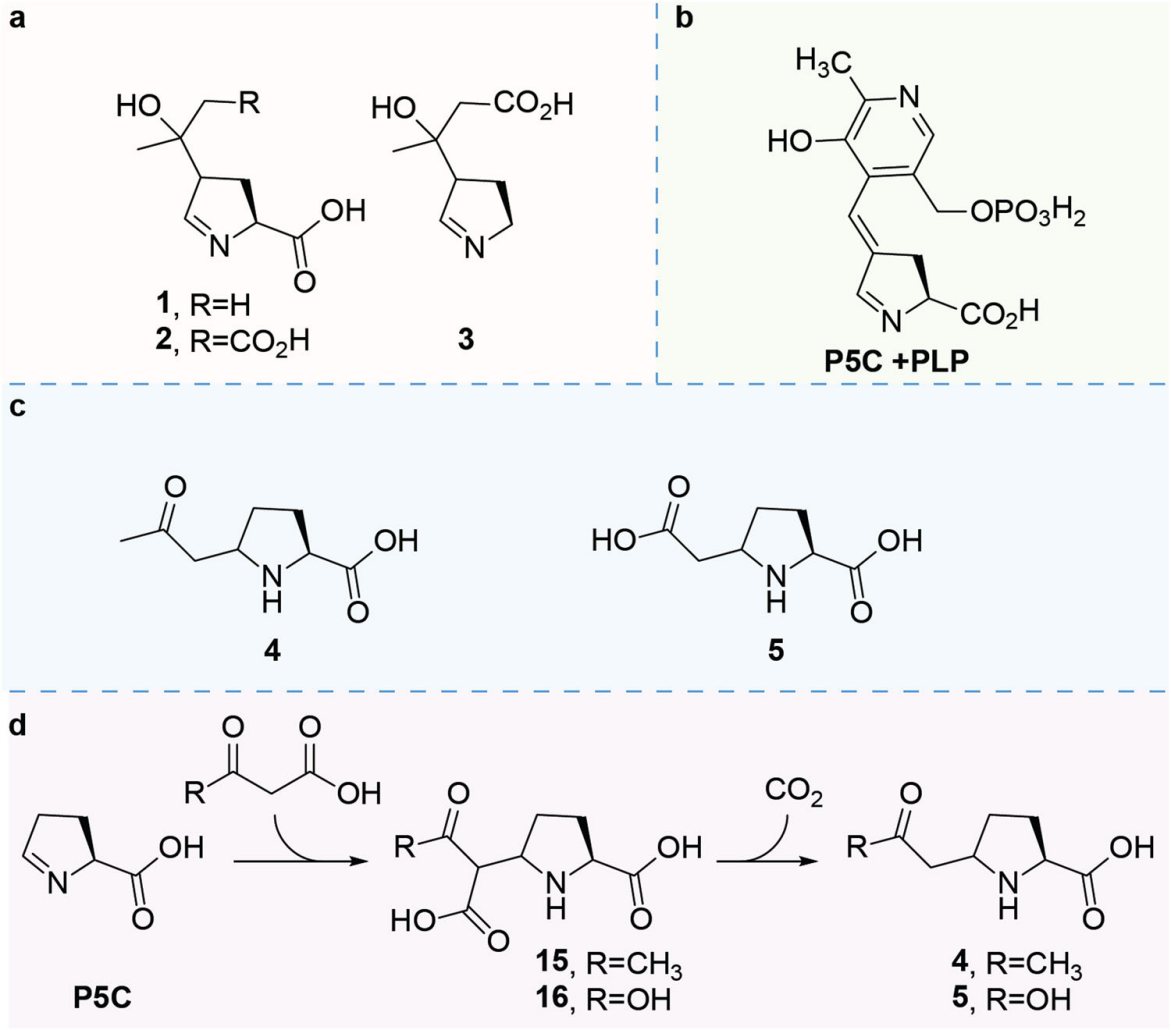
Biomarkers identified for HPII. **a** Proposed molecular structure of the original detected reaction product of P5C with acetoacetic acid^6^. **b** Previously characterized adduct of P5C with PLP. **c** Biomarkers identified in this work. **d** Pathway of formation of the detected biomarkers.

The spontaneous reaction of P5C with acetoacetic acid has been previously noted by the ref. ^6^. In that work, the structural assignment of the metabolites resulting from the incubations of P5C with acetoacetic acid were based on GC-MS/MS after derivation of the reaction mixture with trimethylsilane (TMS). The authors propose a resulting structure of the P5C reaction with acetoacetic acid, before and after decarboxylation, based on an analogy with the reaction of P5C with PLP (Fig. 5b), leading to structures **1-3** (Fig. 5a).

Here, using reference standards characterized by both NMR and IRIS, we identified structures **4** and **5** as HPII-associated metabolites (Fig. 5c) that have potential diagnostic value. The formation of these biomarkers proceeds via addition of 1,3-dicarbonyl compounds to the accumulating cyclic imine, followed by decarboxylation (Fig. 5d). The structures identified in this study for the incubation of P5C with acetoacetic acid, and its carboxylic acid containing intermediate **15**, have the same molecular formulae as those proposed by ref. ^6^. In addition, they contain an equal number of nucleophilic groups amenable to TMS-derivatization. Moreover, during the preparative synthesis of the biomarkers, by incubation of P5C with 1,3-dicarbonyl compounds, we found no spectroscopic evidence of the formation of the structures proposed by ref. ^6^ (Fig. 5a). If these structures were formed, we would expect to observe a unique shift downfield corresponding to the imine proton, as was observed for the adducts of P5C with PLP^5^. We did not observe such evidence for the reaction products with acetoacetic acid. Taken together, it is likely that we detected the same adducts of acetoacetic acid and P5C as described by ref. ^6^. but have now correctly annotated the molecular structure (Fig. 5c). In addition, the formation of the adduct of malonic acid with P5C (Figs. 5c, **5**) indicates a general reactivity of the cyclic imine P5C with other biochemically relevant 1,3-dicarbonyl compounds. Furthermore, 2-OPC (**4**) is present in the body fluids of ketotic patients as the increased acetoacetic acid concentration in their plasma and urine can spontaneously react with small amounts of P5C, leading to the formation of 2-OPC (**4**). A similar spontaneous reaction has been observed in patients with pyridoxine-dependent epilepsy, where P6C reacts spontaneously with the available acetoacetic acid and malonic acid ^10^.

These metabolites may have an impact on the diagnosis and follow-up of patients with HPII as well. By including 2-OPC (**4**) and CPC (**5**) as plasma HPII biomarkers in a diagnostic IEM screening panel, we can readily distinguish between different causes of hyperprolinemia using our NGMS methodology. Additional to HPII, hyperprolinemia type I (HPI, OMIM 239500) is also a genetic condition that is biochemically characterized by proline accumulation in body fluids. However, as the enzyme deficiency in HPI is located one step upstream in proline degradation compared to HPII, in the enzyme proline oxidase, P5C does not accumulate in HPI. HPI can therefore directly be distinguished from HPII by the absence of P5C and its adduct biomarkers in body fluids. The detection of P5C, however, requires derivatization for accurate detection^17,18^. Therefore, an HPII diagnosis can be made more effectively, with simultaneous detection of proline and the metabolites 2-OPC and CPC elucidated in this work. However, the biomarker 2-OPC does not discriminate between HPII and ketotic patients (See SI Figure S1). CPC and the two HPII biomarkers, 2-pyrrolyl-glycine and pyrrole-2-carboxylic acid, on the other hand, do discriminate between HPI, HPII, and ketosis. Regarding the implications of our findings for the pathophysiological understanding of HPII, it is interesting to note that for PDE-ALDH7A1, the analogous P6C-adduct biomarkers were found to have epileptogenic potential in a zebrafish model system and could also be detected in brain tissue of PDE-ALDH7A1 patients. Though out of scope for this current work, future studies are envisioned to also evaluate the possible direct involvement of the P5C-adducts in the etiology of epilepsy in HPII.

Using NGMS in combination with IRIS, we have successfully detected and annotated the molecular structure of metabolites accumulating in HPII. The preliminary annotation of the detected features, based on matching to theoretically predicted IR spectra generated for the candidate structures (postulated on the basis of both MS/MS and the structural information gained from the infrared spectra of the features) limits the number of synthetic standards required. Using this approach, we have identified and characterized two sets of diastereomeric biomarkers, resulting non-enzymatically from the reaction of the 1,3-dicarbonyl compounds malonic and acetoacetic acid with the accumulating cyclic imine P5C in patients of HPII. The identification of the molecular structures of the biomarkers, and their likely biochemical origins, may lead to a better understanding of the pathophysiology of the disease and can lead to an improved diagnosis of HPII.

## Methods

### Sample preparation

For IRIS analysis, 1000 µl of 0.125% [v/v] formic acid in 95:5 [v/v] ACN:deionized water was added to 100 µl of urine and mixed with a vortex mixer for 15 s. The samples were centrifuged at 18,600 × *g* for 20 min at 4 °C. The supernatant was transferred to autosampler vials and used for HILIC-IRIS analysis. Reference standards were analyzed as 10 µM solutions in ACN/deionized water 95:5 [v/v].

### Patients

The body fluid samples described in this paper were from three HPII patients from two independent families with the classical clinical phenotype of HPII, including mental retardation and epilepsy. Three samples were included from a single HPI patient with epilepsy and motor retardation. The proline concentration was repeatedly found highly increased in the body fluids of this patient while the other HPII mentioned in this paper were not increased. In the HPII samples, proline and hydroxyproline were found to increase with dedicated amino acid analysis. Evidence for the accumulation of the HPII biomarker pyrrole-2-carboxylic acid and its glycine ester, 2-pyrrolyl-glycine, was obtained with NGMS. Control persons and ketotic patients in this study were in the same age range as the HPI and II patients.

### NGMS

NGMS was performed as described previously^10,16^. In short, analyses were performed using an Agilent 1290 UHPLC in combination with an Agilent 6545 QTOF mass spectrometer. Chromatographic separations were achieved on an Acquity HSS T3 (C18, 2.1 × 100 mm, 1.8 μm) column (Waters) operating at 40 °C. The analytical batches contained patient samples, validation plasma pool, and analytical quality control (QC) samples to check for the integrity of the automated data analysis pipeline.

### Infrared ion spectroscopy

IRIS experiments were performed using a quadrupole ion trap mass spectrometer (Bruker, AmaZon Speed ETD). The hardware modifications made to this instrument to enable IR spectroscopy and the synchronization of the ion trap with the infrared laser is described elsewhere^19^. HILIC separations were performed using a Bruker Elute SP HPLC system and a Waters Acquity amide column (100 × 2.1 mm i.d., 1.7 μm particles, 130 Å pore size) held at 30 °C. The mobile phase consisted of 10 mM ammonium formate and 0.125% [v/v] formic acid in 95:5 [v/v] ACN:deionised water (mobile phase A) and 50:50 [v/v] ACN:deionised water (mobile phase B). A flow rate of 0.5 ml/min and an injection volume of 10 μl was used. The separation method consisted of an initial time of 1 min at 100% A, followed by a gradient to 100% B in 10 min, and hold at 100% B for 1 min. An equilibration time of 8 min was used. Fractions of the metabolites were collected using a two-position six-port switching valve controlled by the ion trap and an 80 μl sample loop installed on this valve. Most of the HPLC eluent was diverted to waste, but as soon as a fraction of interest arrived at the valve, the valve was switched and the fraction was collected in the sample loop. Following another valve switch, the fraction was slowly infused by a syringe pump (120 μl/h) into the ion trap for IRIS analysis.

IR spectra were recorded using the FELIX infrared free-electron laser, which produced IR radiation in the form of ∼10 μs macropulses of 50–150 mJ at a 10 Hz repetition rate (bandwidth ∼0.4% of the center frequency). Mass-isolated ions were irradiated inside the ion trapping region where resonant absorption of the IR light by the ions leads to an increase in internal energy and, after reaching the dissociation threshold, to photodissociation. This was observed by recording a fragmentation mass spectrum after irradiation at each wavelength point. Each fragmentation spectrum was constructed from 4–8 averaged spectra. IR spectra were constructed by plotting the fractional dissociation (IR yield = ΣI(fragment ions)/ΣI(parent + fragment ions)) as a function of IR laser frequency. The IR yield was linearly corrected for frequency-dependent variations in laser pulse energy^20^ and the IR frequency was calibrated using a grating spectrometer.

### Quantum-chemical calculations

To predict the IR spectra of candidate structures, DFT calculations were performed using the Gaussian16 software package^21^. Molecular structures were manually defined and optimized at the B3LYP/6 − 31 + +G(d,p) level of theory. This was followed by a vibrational frequency calculation within the harmonic approximation. Calculated spectra were scaled using a linear scaling factor of 0.975 and broadened using a Gaussian line shape function (20 cm^−1^ full width at half maximum) to facilitate comparison with the experimental IR spectra.

### NMR spectroscopy

^1^H and ^13^C NMR spectra were recorded on a Bruker AVANCE III (500 MHz ^1^H, 126 MHz ^13^C) equipped with a Bruker Prodigy BB cryoprobe or a Bruker AVANCE III (400 MHz ^1^H, 100 MHz ^13^C) in the solvent indicated at room temperature. Chemical shifts are reported in δ (ppm) units relative to the solvent residual peak. For ^1^H NMR spectra, the following abbreviations are used to describe multiplicities: s (singlet), d (doublet), t (triplet), dd (double doublet), and m (multiplet). Coupling constants are reported in Hertz (Hz) as a *J* value.

### Chemicals

All solvents were reagent grade and, when necessary, were purified and dried by standard methods. All commercially purchased reagents were used without further purification as delivered from the corresponding companies.

### Synthesis

#### *Tert-*butyl 5-oxopyrrolidine-2-carboxylate^22^

Perchloric acid (9.5 mL, 95 mmol, 60%) was added dropwise to a suspension of 5-oxopyrrolidine-2-carboxylic acid (30.0 g, 0.39 mol) in *tert*-butyl acetate (220 mL, 1.64 mol) and the mixture was stirred at rt for 24 h. Then another portion of Perchloric acid (9.5 mL, 95 mmol, 60%) was added and the stirring continued for 20 h. The reaction mixture was quenched with solid NaHCO_3_ diluted with H_2_O and extracted thrice with EtOAc. The combined organic layers were washed with brine and dried over MgSO_4_, filtered, and evaporated to dryness. Which resulted in a white solid with a yield of 46% (30.5 g, 0.18 mol). R_*f*_ = 0.19 (4:1, EtOAc:heptane); ^1^H NMR (400 MHz, CDCl_3_) δ 6.26 (s, 1H), 4.18–4.10 (m, 1H), 2.51–2.27 (m, 3H), 2.25–2.12 (m, 1H), 1.49 (s, 9H); ^13^C NMR (101 MHz, CDCl_3_) δ 177.7, 171.0, 82.9, 55.9, 29.3, 28.0, 24.9; HRMS (ESI^+^): calcd for C_9_H_15_NNaO_3_ [M + Na]^+^: 208.0950, found 208.0955.

#### Di-*tert*-butyl 5-oxopyrrolidine-1,2-dicarboxylate^22^

DMAP (0.66 g, 5.4 mmol) and Boc_2_O (26 g, 0.12 mol) were added to a solution of *tert*-butyl 5-oxopyrrolidine-2-carboxylate (20 g, 0.11 mol) in MeCN (180 ml). The solution was stirred at rt for 72 h. The solvent was then removed under reduced pressure, the residue taken up in an EtOAc/CyHex mixture (400 ml, 1:1), filtered over silica, washed twice with EtOAc/Hept (100 mL, 4:1), and evaporated to dryness, which resulted in an off-white solid with a yield of 97% (30 g, 0.11 mol). R_*f*_ = 0.68 (4:1, EtOAc:heptane); ^1^H NMR (400 MHz, CDCl_3_) δ 4.47 (dd, *J* = 9.5, 2.6 Hz), 2.60 (m), 2.46 (m), 2.28 (ddt, *J* = 13.4, 10.5, 9.4 Hz), 2.06 – 1.93 (m), 1.50 (s), 1.48 (s); ^13^C NMR (101 MHz, CDCl_3_) δ 173.5, 170.4, 150.7, 84.0, 82.3, 59.6, 31.2, 28.0, 27.9, and 21.7.

#### Di- *tert*- butyl (2 *s*)-5-hydroxypyrrolidine - 1,2 –dicarboxylate^23^

To a solution of di-*tert*-butyl 5-oxopyrrolidine-1,2-dicarboxylate (3.0 g, 10.5 mmol) in dry THF (50 mL) at −78 °C was added slowly a 1 M DIBAL-H solution (21 ml, 21 mmol). After 3 h stirring at −78 °C, the excess reagent was destroyed through the addition of isopropanol. Then sat. aq. sodium-potassium l-tartrate (Rochelle salt) was added and the reaction mixture was allowed to slowly warm to rt overnight. The mixture was partitioned between H_2_O and EtOAc (5:1). The aqueous phase was further extracted twice with EtOAc. The collected organic layers were dried over MgSO_4_ and concentrated to dryness. Purification by column chromatography (0–>60% EtOAc in heptane) resulted in a colorless oil with a yield of 44.5% (1.3 g, 4.7 mmol). R_*f*_ = 0.35 (1:1 EtOAc:heptane); ^1^H NMR (500 MHz, CDCl_3_, mixture of diastereoisomers) δ 5.65 (dd, *J* = 6.0, 2.4 Hz, 0.6H), 5.60 (dt, *J* = 5.5, 2.9 Hz, 0.7H), 5.54 (dd, *J* = 5.8, 2.4 Hz, 0.2H), 5.51–5.45 (m, 0.3H), 4.24 (dd, *J* = 8.8, 1.3 Hz, 0.8H), 4.15 (dd, *J* = 8.5, 6.7 Hz, 0.7H), 3.57 (t, *J* = 2.0 Hz, 0.5H), 3.48 (d, *J* = 3.4 Hz, 0.7H), 3.12 (d, *J* = 5.0 Hz, 0.3H), 2.88 (t, *J* = 2.0 Hz, 0.2H), 2.52–2.41 (m, 0.5H), 2.25 (dtd, *J* = 13.2, 8.3, 5.0 Hz, 1H), 2.17–2.01 (m, 2H), 2.04–1.83 (m, 2H), 1.74 (s, 3H), 1.48–1.45 (m, 18H), 1.34–1.24 (m, 1H); ^13^C NMR (126 MHz, CDCl_3_, mixture of diastereoisomers) δ 172.2, 171.6, 154.4, 154.0, 123.0, 129.7, 128.4, 82.6, 82.32, 82.29, 81.3, 81.1, 80.78, 80.75, 60.04, 59.95, 59.91, 33.6, 32.2, 31.7, 30.9, 28.5, 28.4, 28.29, 28.28, 28.0, 27.94, 27.89, 27.1, and 26.9.

#### Δ-1-pyrroline-5-carboxylate.HCl (P5c)^24^

Di-*tert*-butyl (2 S)-5-hydroxypyrrolidine-1,2-dicarboxylate (750 mg, 2.6 mmol) was dissolved in dioxane (10 mL) and HCl (20 mL, 6 molar) was added and was stirred for 1 h. The reaction mixture was concentrated in vacuo and used as is.

#### Benzyl(2 *s*)-1-benzyl-5-(2-(benzyloxy)-2-oxoethyl) pyrrolidine-2-carboxylate

A solution of malonic acid (108 mg) in H_2_O (5 mL) was neutralized to pH 7 with solid NaOH. To this solution was added the mixture of P5C.HCl (390 mg, 2.6 mmol) while keeping the pH between 6 and 7 with the addition of NaOH (2 M). After the solution (final pH = 6.5) was stirred for 4 days, it was acidified to pH = 1 and refluxed for 30 min, and subsequently concentrated in vacuo. The resulting residue was redissolved in DMF (10 mL) and cooled to 0 °C. Solid K_2_CO_3_ (717 mg, 5.2 mmol) was added followed by the addition of BnBr (0.61 mL, 5.1 mmol). The resulting mixture was stirred overnight and more BnBr (0.2 mL, 2.0 mmol) and K_2_CO_3_ (190 mg, 1.37 mmol) were added and the solution was stirred for an additional 4 h. The solution was concentrated *in vacuo*, the residue was taken up in EtOAc (10 mL), washed with H_2_O (5 mL) and brine (5 mL), dried over MgSO_4_, and concentrated in vacuo. Purification by column chromatography (30% EtOAc in heptane) followed by (5% Et_2_O in toluene) resulted in the separation of the diastereoisomers, obtained in a yield of 12% for *cis* (128.6 mg, 0.29 mmol), and 10% for *trans* (115.5 mg, 0.26 mmol). *Cis*: ^1^H NMR (400 MHz, CDCl_3_) δ 7.41 – 7.29 (m, 8H), 7.33 – 7.24 (m, 5H), 7.27 – 7.16 (m, 2H), 5.10 (s, 2H), 5.01 – 4.90 (m, 2H), 3.92 (d, *J* = 13.9 Hz), 3.78 (d, *J* = 13.9 Hz), 3.49 – 3.41 (m, 1H), 3.32 – 3.23 (m, 1H), 2.68 (dd, *J* = 15.2, 4.4 Hz), 2.44 (dd, *J* = 15.2, 8.9 Hz), 2.09– 1.86 (m, 3H), 1.78 – 1.65 (m, 1H); ^13^C NMR (100 MHz, CDCl_3_) δ 174.2, 172.0, 138.3, 136.0, 129.2, 128.53, 128.48, 128.2, 128.14, 128.10, 128.09, 127.1, 66.19, 66.16, 66.1, 62.5, 61.3, 57.7, 40.5, 30.8, 28.4; MS (ESI^+^): calcd for C_28_H_30_NO_4_ [M + H]^+^: 444.22, found 444.24. *Trans*: ^1^H NMR (400 MHz, CDCl_3_) δ 7.42 – 7.28 (m, 10H), 7.27 – 7.16 (m, 5H), 5.23 – 5.02 (m, 4H), 3.95 (d, *J* = 13.5 Hz, 1H), 3.77 – 3.67 (m, 2H), 3.61 (dd, *J* = 8.2, 1.6 Hz, 1H), 2.68 (dd, *J* = 14.6, 3.9 Hz, 1H), 2.40 (dd, *J* = 14.6, 8.7 Hz, 1), 2.33 – 2.21 (m, 1H), 2.15 – 2.03 (m, 1H), 1.85 – 1.74 (m, 2H); ^13^C NMR (100 MHz, CDCl_3_) δ 173.5, 171.4, 139.2, 136.0, 128.58, 128.55, 128.5, 128.33, 128.27, 128.25, 128.22, 128.21, 127.0, 66.2, 65.9, 62.9, 58.6, 52.7, 39.8, 29.4, 27.7; MS (ESI^+^): calcd for C_28_H_30_NO_4_ [M + H]^+^: 444.22, found 444.24.

#### Benzyl (2 *s*,5*r*)-1-benzyl-5-(2-oxopropyl)pyrrolidine-2-carboxylate

A solution of freshly prepared acetoacetic acid (750 mg) by quantitative acidic deprotection of *tert*-butyl acetoacetate, in H_2_O was neutralized to pH 7 with NaOH. To this solution was added freshly prepared P5C (214 mg, 1.43 mmol), maintaining the pH between 6 and 7 with the concomitant addition of 1 M NaOH. The mixture was left standing at rt for 2 days, acidified to pH = 1 with 1 M HCl and brought to reflux for 30 min. The mixture was concentrated *in vacuo* followed by removal of the salts with a hot filtration from ethanol. The filtrate was concentrated in vacuo and dissolved in DMF (10 mL). Solid K_2_CO_3_ (1.2 g, 8.6 mmol), as well as BnBr (0.85 mL, 7.2 mmol), were added and the reaction mixture was stirred overnight. The mixture was concentrated in vacuo, the residue was taken up in EtOAc and the organic layer was washed with H_2_O thrice and brine, dried over MgSO_4_, and concentrated in vacuo. Purification by column chromatography (5% Et_2_O in toluene) resulted in the isolation of only the *cis* stereoisomer as a sufficiently pure product, which was obtained as a white solid in a 6% yield (32.5 mg, 92 µmol). R_*f*_ = 0.6 (1:1 EtOAc:heptane); ^1^H NMR (500 MHz, CDCl_3_) δ 7.43 – 7.17 (m, 10H), 5.07 – 4.90 (m, 2H), 3.92 – 3.74 (m, 2H), 3.44 (dd, *J* = 7.9, 6.4 Hz, 1H), 3.33 – 3.24 (m, 1H), 2.70 (dd, *J* = 16.7, 4.4 Hz, 1H), 2.50 (dd, *J* = 16.6, 8.3 Hz, 1H), 2.13 – 2.01 (m, 5H), 1.98 – 1.88 (m, 1H), 1.64 – 1.56 (m, 1H); ^13^C NMR (126 MHz, CDCl_3_) δ 208.4, 174.4, 138.7, 136.0, 129.3, 128.6, 128.26, 128.24, 127.2, 66.32, 66.28, 60.8, 58.2, 49.6, 31.04, 30.97, and 28.6.

#### General procedure A: hydrogenation

A solution of the benzylated compound (1.0 eq) in methanol (40 mM) was purged with inert gas, 10% Pd/C (10 mol%) was added, and the atmosphere was exchanged for H_2_. The suspension was stirred at rt under an H_2_ atmosphere for 20 h. The flask was evacuated and purged with nitrogen and the mixture was filtered through Celite with a 50 mL methanol rinse. The filtrate was concentrated in vacuo and lyophilized.

#### (2 *s*,5*r*)-5-(carboxymethyl)pyrrolidine-2-carboxylic acid

Benzyl(2 *s*,5*r*)-1-benzyl-5-(2-(benzyloxy)-2-oxoethyl) pyrrolidine-2-carboxylate (95 mg, 210 μmol) was hydrogenated according to procedure A, resulting in a fluffy white solid in quantitative yield (24 mg, 138 μmol) after lyophilization. ^1^H NMR (500 MHz, D_2_O) δ 4.12 (dd, *J* = 9.3, 4.7 Hz, 1H), 3.89 (ddd, *J* = 11.9, 8.6, 6.0 Hz, 1H), 2.91 – 2.75 (m, 2H), 2.28 (m, 1H), 2.22 – 2.08 (m, 2H,), 1.72 – 1.60 (m, 1H); ^13^C NMR (126 MHz, D_2_O) δ 175.0, 174.4, 61.2, 57.2, 36.2, 28.8, 28.1; HRMS (ESI^+^): calcd for C_7_H_12_NO_4_ [M + H]^+^ 174.0761, found 174.0765.

#### (2 *s*,5 *s*)-5-(carboxymethyl)pyrrolidine-2-carboxylic acid

Benzyl(2 *s*,5 *s*)-1-benzyl-5-(2-(benzyloxy)-2-oxoethyl) pyrrolidine-2-carboxylate (128.6 mg, 289.9 μmol) was hydrogenated according to procedure A, resulting in a fluffy white solid in 65% yield (49 mg, 138 μmol) after lyophilization which, after lyophilization contained a minor aromatic impurity (<10% based on NMR). ^1^H NMR (500 MHz, D_2_O) δ 4.07 (t, *J* = 8.3 Hz, 1H), 3.83 (dq, *J* = 9.5, 6.5 Hz, 1H), 2.62 – 2.49 (m, 2H), 2.38 (dtd, *J* = 12.9, 8.1, 3.6 Hz, 1H), 2.14 (dtd, *J* = 13.5, 6.9, 3.7 Hz, 1H), 1.94 (ddt, *J* = 13.1, 9.7, 7.8 Hz, 1H), 1.67 (dtd, *J* = 13.1, 9.5, 7.7 Hz, 1H); ^13^C NMR (126 MHz, D_2_O) δ 178.1, 175.3, 138.9, 60.7, 57.8, 38.2, 30.0, and 28.6; HRMS (ESI^+^): calcd for C_7_H_12_NO_4_ [M + H]^+^: 174.0761, found 174.0765.

#### (2 *s*,5*r*)-5-(2-oxopropyl)pyrrolidine-2-carboxylic acid

Benzyl (2 *s*,5*r*)-1-benzyl-5-(2-oxopropyl)pyrrolidine-2-carboxylate (30 mg, 85 μmol) was hydrogenated according to procedure A, substituting MeOH for MeCN. This resulted in a fluffy white solid obtained in quantitative yield (15 mg, 85 μmol). ^1^H NMR (500 MHz, D_2_O) δ 4.09 (dd, *J* = 9.5, 4.2 Hz, 1H), 3.88 (tt, *J* = 10.0, 5.6 Hz, 1H), 3.14 (dd, *J* = 19.2, 4.3 Hz, 1H), 3.05 (dd, *J* = 19.2, 9.0 Hz, 1H), 2.27 – 2.20 (m, 1H), 2.18 (d, *J* = 0.6 Hz, 3H), 2.13 (tdd, *J* = 9.7, 6.6, 3.5 Hz, 2H), 1.66 – 1.57 (m, 1H); ^13^C NMR (126 MHz, D_2_O) δ 210.9 (HMBC), 61.3, 56.2, 44.6, 29.1, 28.7, and 28.2; LC-HRMS (ESI^+^): calcd for C_8_H_14_NO_3_ [M + H]^+^: 172.0968, found 172.0964.

#### di-*ter*t-butyl (2 *s*)-5-methoxypyrrolidine-1,2-dicarboxylate

Di-*tert*-butyl (2 *s*)-5-hydroxypyrrolidine-1,2-dicarboxylate (105.5 mg, 367.1 μmol) was dissolved in methanol (5 mL) and catalytic amount of p-toluenesulfonic acid monohydrate (3.3 mg, 17 μmol) was slowly added. The reaction was stirred for 48 h. The reaction mixture was diluted with EtOAc (20 mL). The organic layer was washed thrice with saturated aqueous NaHCO_3_ and dried over MgSO_4_, filtered, and evaporated under reduced pressure. R_*f*_ = 0.45 (3:7 EtOAc:Heptane), ^1^H NMR (400 MHz, CDCl_3_, diastereoisomers, signal of *tert-*butyl set as 18) δ 5.31 (dd, *J* = 11.2, 4.6 Hz, 0.6H), 5.20 (d, *J* = 4.7 Hz, 0.2H), 5.15 (d, *J* = 4.9 Hz, 0.2H), 4.27 – 4.09 (m, 1H), 3.45 (s, 1H), 3.42 (s, 1.4H), 3.37 (s, 0.4H), 2.50 – 2.24 (m, 0.7H), 2.21 – 2.07 (m, 0.2H), 2.01 – 1.81 (m, 1.5H), 1.61 (s, 1.8H), 1.55 – 1.44 (m, 18H); ^13^C NMR (101 MHz, CDCl_3_) δ 202.3, 171.8, 89.37, 89.33, 88.5, 81.0, 80.4, 60.4, 60.0, 59.9, 56.2, 55.8, 55.3, 32.3, 31.1, 29.9, 28.32, 28.25, 28.03, 28.01, 27.98, 27.91, and 27.1; HRMS (ESI^+^): Calcd. for C_15_H_27_NNaO_5_ [M + Na]^+^: 324.1781, found 324.1787.

#### Di-*tert*-butyl (2s)-5-(2-oxopropyl)pyrrolidine-1,2-dicarboxylate

To a solution of di-*tert*-butyl (2 s)-5-methoxypyrrolidine-1,2-dicarboxylate (106.5 mg, 353.4 μmol) in dry acetonitrile (10 mL), *tert*-butyldimethyl(prop-1-en-2-yloxy)silane (118.2 mg, 685.9 μmol) was added and the mixture was cooled to −30 °C. Tin(II) trifluoromethanesulphonate (75.1 mg, 180 μmol) was added and the mixture was stirred at rt for 72 h. The mixture was diluted with EtOAc (10 mL) and quenched with sat. aq. NaHCO_3_ (5 mL). The layers were separated and the organic layer was dried over MgSO_4_, filtered, and evaporated under reduced pressure. The resulting residue was purified by column chromatography (0 → 30% EtOAc in heptane) Which resulted in a yield of 25% (89.1 μmol, 29.2 mg). R_*f*_ = 0.36 (3:7 EtOAc:heptane); ^1^H NMR (500 MHz, CDCl_3_, diastereomers, signal of *tert-*butyl set as 18): δ 4.43 – 4.35 (m, 0.4H), 4.35 – 4.18 (m, 0.7H), 4.18 – 4.11 (m, 0.8H), 3.39 (dd, *J* = 17.1, 3.4 Hz, 0.5H), 3.16 (dd, *J* = 16.0, 3.4 Hz, 0.5H), 2.96 (dd, *J* = 16.0, 3.0 Hz, 0.3H), 2.65 (dd, *J* = 16.9, 9.7 Hz, 0.3H), 2.55 (dd, *J* = 17.1, 10.0 Hz, 0.5H), 2.41 (td, *J* = 15.9, 15.4, 10.1 Hz, 0.5H), 2.18 (s, 5H), 1.96 – 1.87 (m, 0.6H), 1.72 – 1.63 (m, 1H), 1.52 – 1.43 (m, 18H); ^13^C NMR (126 MHz, CDCl_3_, diastereomers) δ 207.6, 172.0, 60.5, 54.5, 54.2, 47.8, 30.0, 28.8 28.44, 28.37, 28.33, 28.03, and 28.01; HRMS (ESI^+^): calcd for C_17_H_29_NNaO_5_ [M + Na]^+^: 350.1943, found 350.1942.

#### (2 *s*,5)-5-(2-oxopropyl)pyrrolidine-2-carboxylic acid TFA

Di-*tert*-butyl (2 *s*,5)-5-(2-oxopropyl)pyrrolidine-1,2-dicarboxylate (20 mg, 61 μmol) was dissolved in a mixture of TFA:H_2_O:TIS (10 ml, 50:45:5). After 1 h, the solvent was evaporated and the remaining TFA was evaporated twice in the presence of toluene and the residue was subsequently lyophilized to obtain the brown waxy substance within a yield of 92% (56.5 μmol, 15.2 mg). R_*f*_ = 0.05 (1:10 MeOH:DCM); ^1^H NMR (400 MHz, D_2_O, diastereomers) δ 4.35 – 4.25 (m, 1H), 4.05 – 3.85 (m, 1H), 3.21 – 2.91 (m, 2H), 2.57– 2.22 (m, 5H), 2.21 – 2.06 (m, 0.5H), 1.88 – 1.67 (m, 1H). ^13^C NMR (101 MHz, D_2_O, diastereomers) δ 210.8, 210.7, 172.7, 90.5, 60.2, 59.7, 56.3, 55.9, 44.4, 44.2, 29.4, 29.09, 29.07, 28.5, 27.7, 27.6; HRMS (ESI^+^): calcd for C_8_H_13_NO_3_ [M + H]^+^: 172.0968, found 172.0974.

### Reporting summary

Further information on research design is available in the Nature Research Reporting Summary linked to this article.

## Supplementary information

Supplementary Information

Description of Additional Supplementary Files

Supplementary Data 1

Supplementary Data 2

Supplementary Data 3

Supplementary Data 4

Supplementary Data 5

Reporting Summary

## Data Availability

Supplementary Figures and NMR spectra are available in the Supplementary Information file. Data underlying the presented MS/MS spectra are available in Supplementary Data 1. Data underlying the experimental IR spectra are available in Supplementary Data 2. Data underlying the theoretical IR spectra and coordinates of the quantum-chemically optimized structures presented in Supplementary Fig. S8 are available in Supplementary Data 3. Data underlying the relative abundance of the m/z 172.0968 in Fig. S1 are available in Supplementary Data 4. Data underlying the HPLC traces in Figs. S2–7 and S9 are available in Supplementary Data 5. Raw research data files are archived locally on data servers and are available upon reasonable request from the corresponding authors (jonathan.martens@ru.nl, thomas.boltje@ru.nl).
