# Supplementary material for: Identification of Δ-1-pyrroline-5-carboxylate derived biomarkers for hyperprolinemia type II
Source: Commun Biol. 2022 Sep 21;5:997. doi: 10.1038/s42003-022-03960-2 (PMC9492674; doi:10.1038/s42003-022-03960-2)
Supplement: Supplementary file 1 — Supplementary Information [file 42003_2022_3960_MOESM1_ESM.pdf]

# Identification of Δ-1-pyrroline-5-carboxylate derived biomarkers characteristic for hyperprolinemia type II

Jona Merx[1±], Rianne E. van Outersterp[2±], Udo F.H. Engelke[3±], Veronique Hendriks[1], Ron A. Wevers[3,4], Marleen C.D.G. Huigen[3,4], Huub W.A.H. Waterval[5], Irene M.L.W. Körver-Keularts[4,5], Jasmin Mecinović[6], Floris P.J.T. Rutjes[1], Jos Oomens[2], Karlien L.M. Coene[3,4,7], Jonathan Martens[2*], Thomas J. Boltje[1*].

[1]*Radboud University, Institute for Molecules and Materials, Synthetic Organic Chemistry, Heyendaalseweg 135, 6525 AJ, Nijmegen, The Netherlands*

[2]*Radboud University, Institute for Molecules and Materials, FELIX Laboratory, Toernooiveld 7, 6525 ED, Nijmegen, the Netherlands*

[3]*Department of Laboratory Medicine, Translational Metabolic Laboratory, Radboud University Medical Center, Geert Grooteplein Zuid 10, 6525 GA Nijmegen, the Netherlands*

[4]*United for Metabolic Disease, UMD, Amsterdam, The Netherlands*

[5]*Department of Clinical Genetics, Maastricht University Medical Center, Maastricht, The Netherlands*

[6]*Department of Physics, Chemistry and Pharmacy, University of Southern Denmark, Campusvej 55, 5230 Odense, Denmark.*

[7]*Department of Clinical Chemistry and Hematology, Elisabeth-TweeSteden Hospital, Tilburg, The Netherlands*

*corresponding author

E-mail: jonathan.martens@ru.nl, thomas.boltje@ru.nl

## Supplementary figures

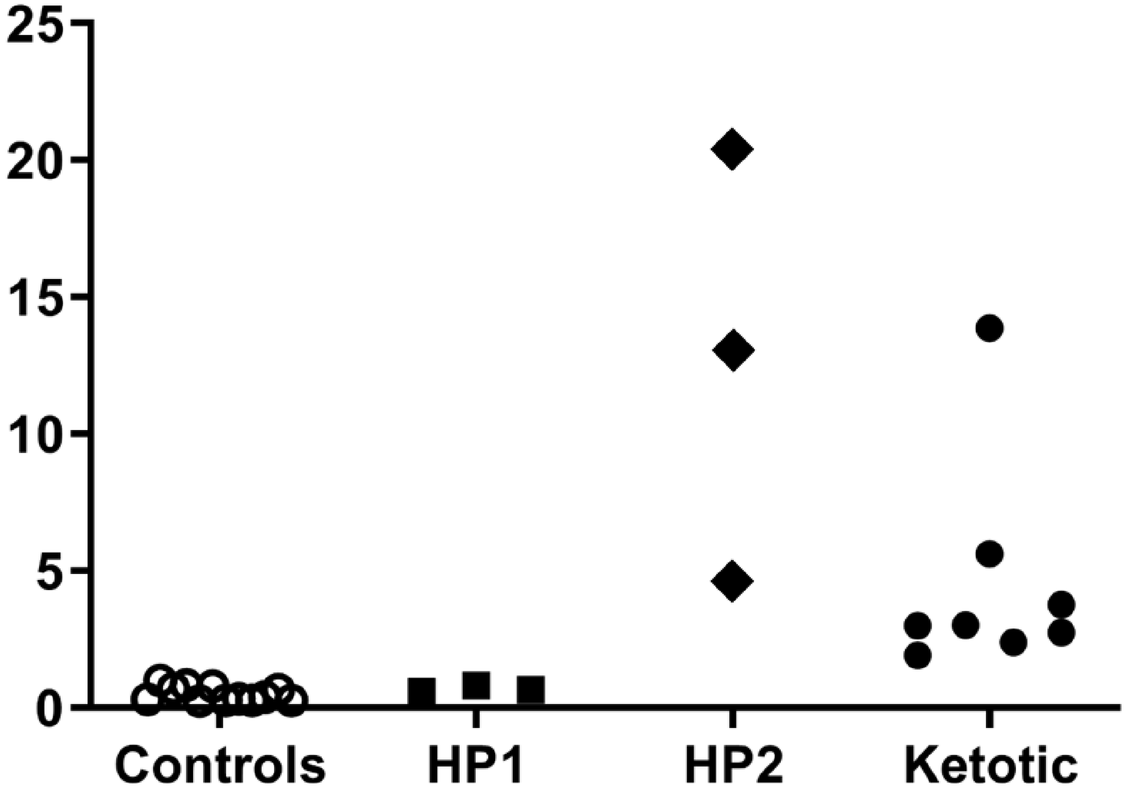


**Figure S1. Relative abundance of the *m/z* 172.0968 (ESI$^+$) ion relative to quality control plasma sample**. Measured in 12 control plasma (open circle, 12 unique samples), HPI plasma (filled square, 3 plasma samples of one HPI patient), HPII plasma (diamonds, 3 samples of 3 patients), and plasma samples of ketotic patients (filled circles, 8 samples of independent patients).

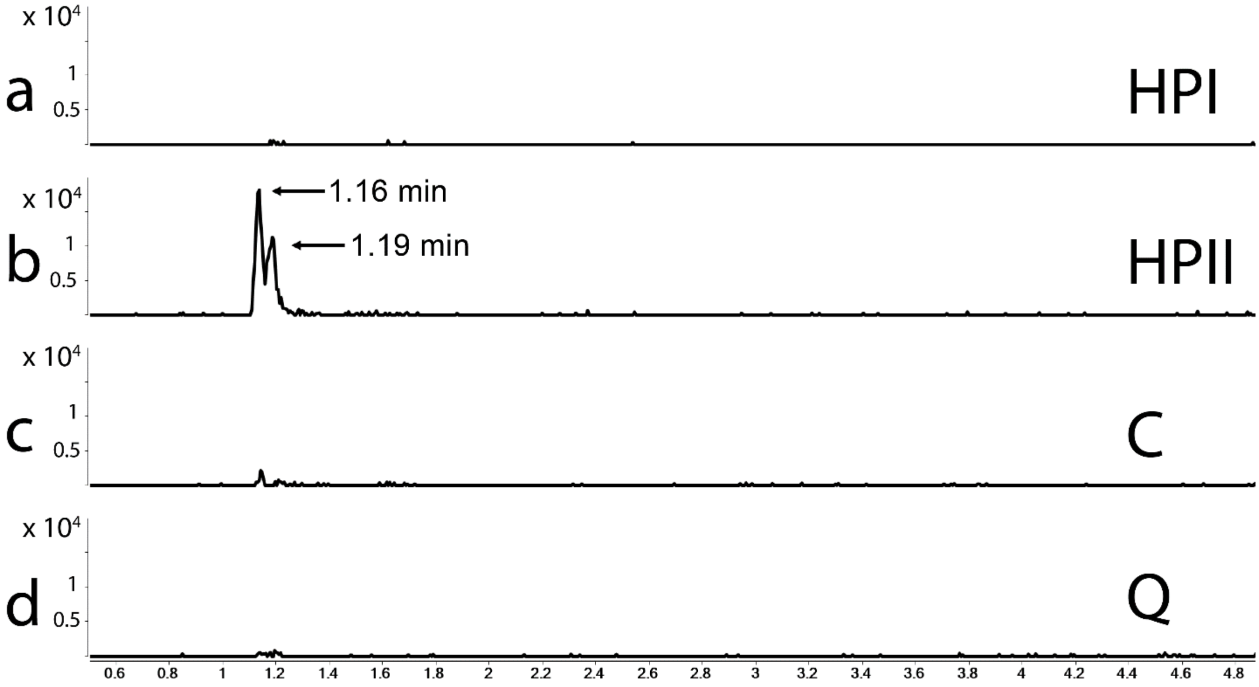


**Figure S2. Extracted ion chromatogram of m/z 170.0823 measured in negative mode. a)** HPI plasma, **b)** HPII plasma, **c)** Control plasma and **d)** Quality control sample

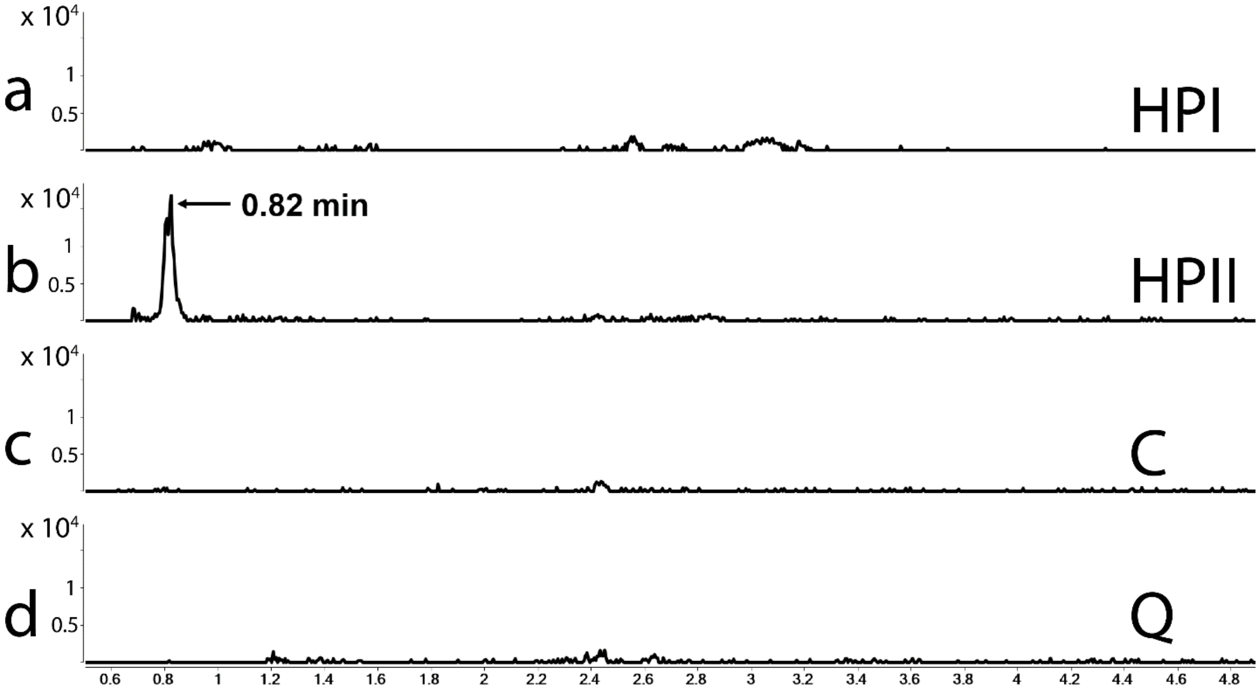


**Figure S3. Extracted ion chromatogram of m/z 172.0615 measured in negative mode. a)** HPI plasma, **b)** HPII plasma, **c)** Control plasma and **d)** Quality control sample

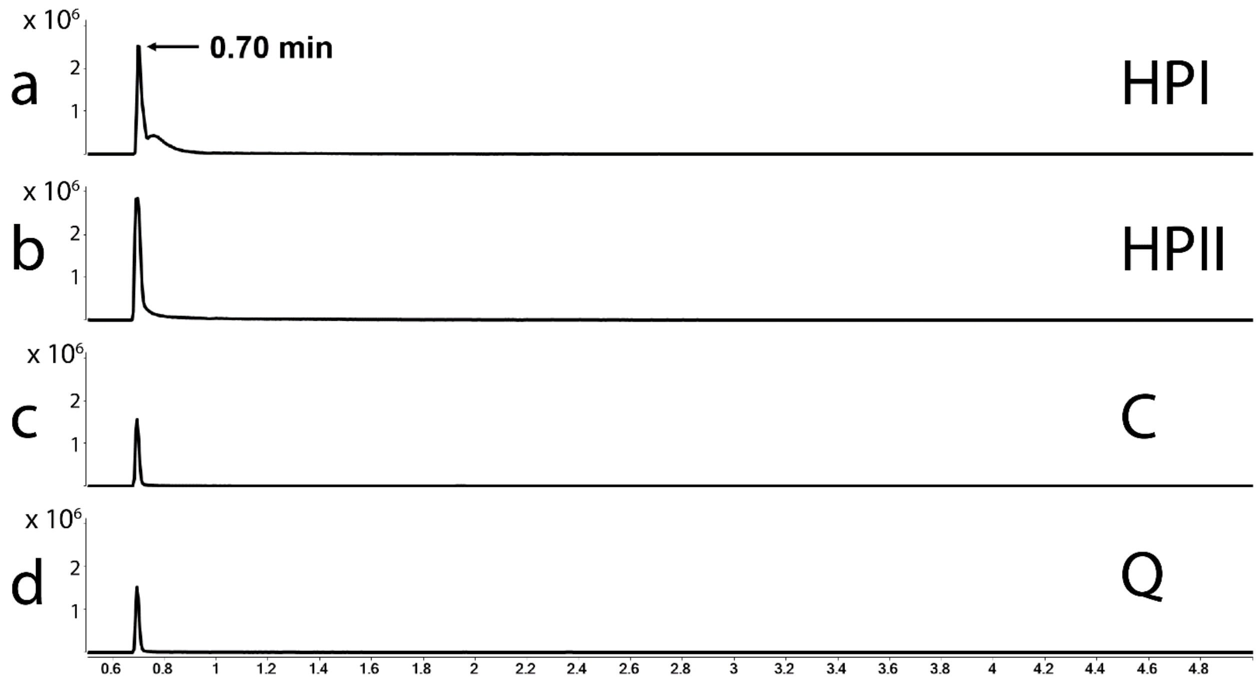


**Figure S4. Extracted ion chromatogram of m/z 117.0739 measured in positive mode. a)** HPI plasma, **b)** HPII plasma, **c)** Control plasma and **d)** Quality control sample

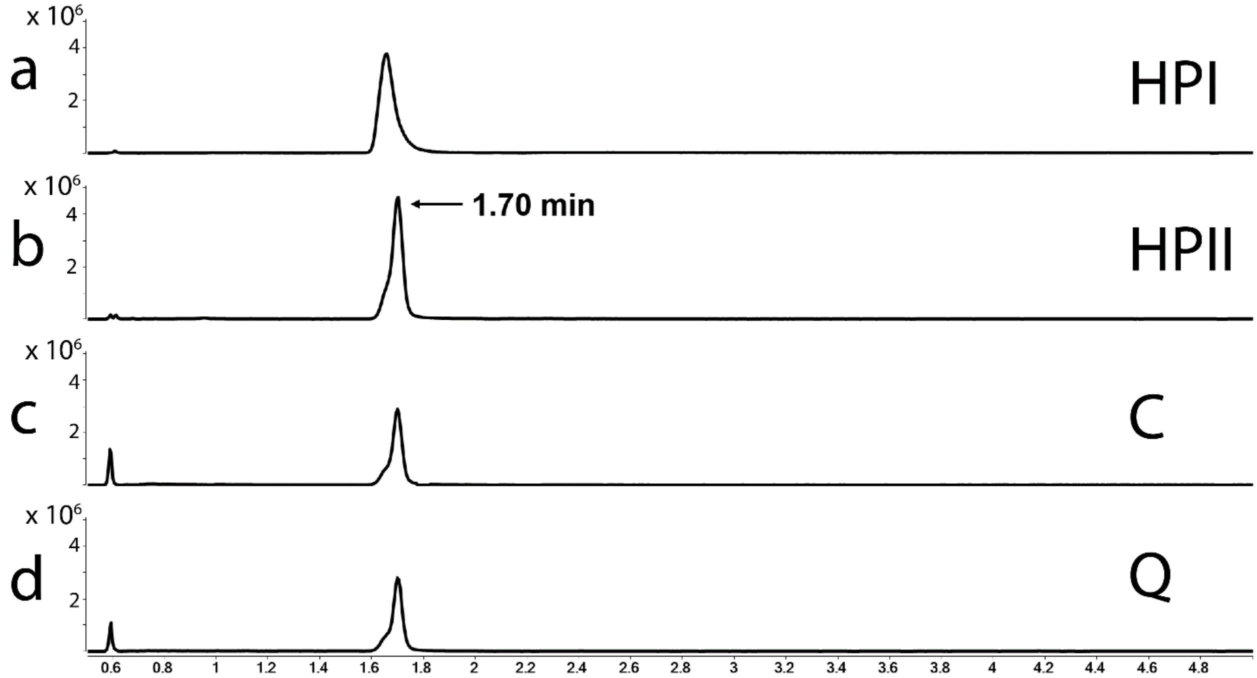


**Figure S5. Extracted ion chromatogram of m/z 130.0499 measured in positive mode. a)** HPI plasma, **b)** HPII plasma, **c)** Control plasma and **d)** Quality control sample

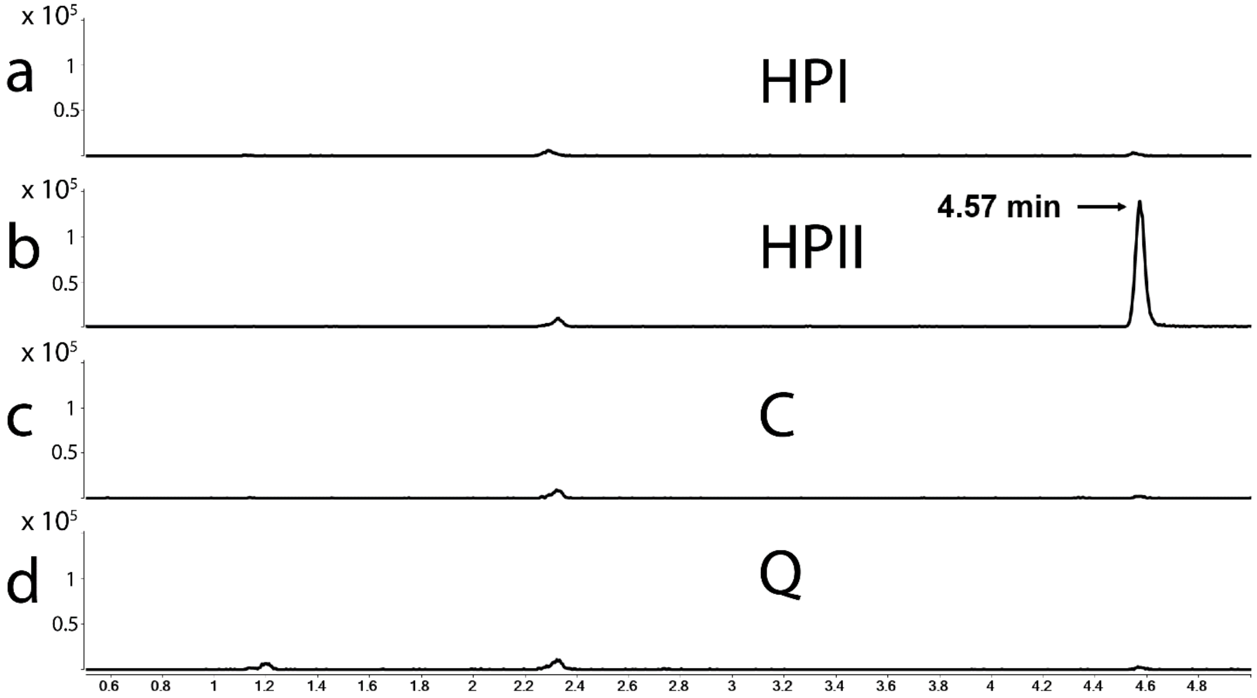


**Figure S6. Extracted ion chromatogram of m/z 110.0248 measured in negative mode. a)** HPI plasma, **b)** HPII plasma, **c)** Control plasma and **d)** Quality control sample

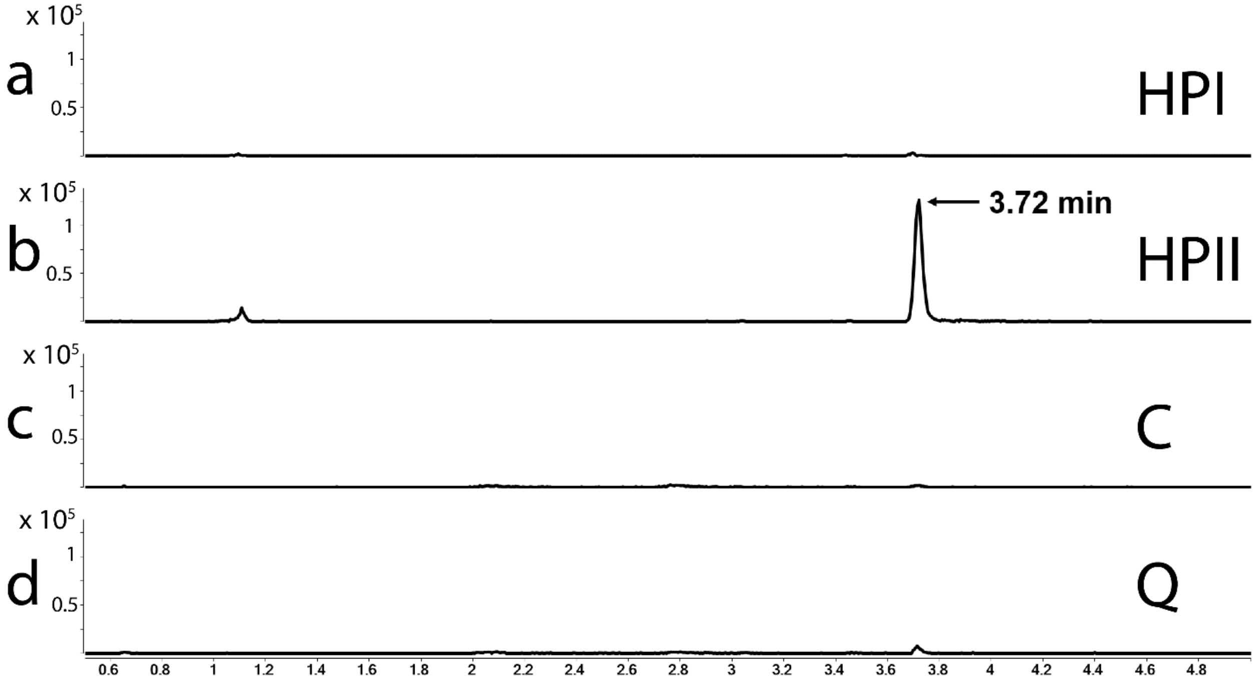


**Figure S7. Extracted ion chromatogram of m/z 167.0456 measured in negative mode. a)** HPI plasma, **b)** HPII plasma, **c)** Control plasma and **d)** Quality control sample

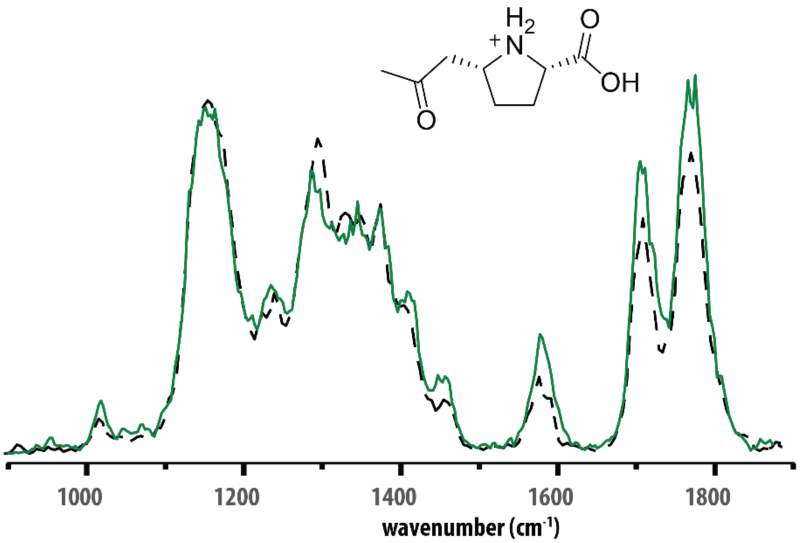


**Figure S8.** Comparison of the IRIS spectra of feature A (dashed black trace) with the single isolated diastereoisomer **4*cis*** (green trace).

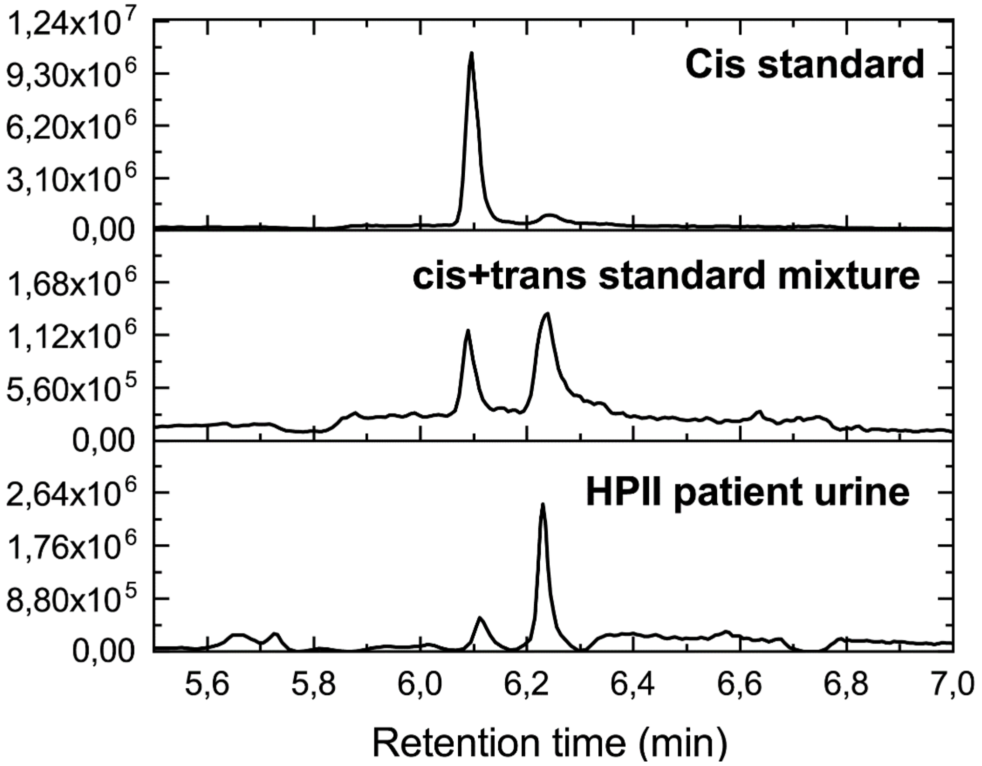


**Figure S9.** Comparison of the retention time on HILIC of the single diastereoisomer **4cis** (top), with the synthetic standards as a diastereo-isomeric mixture **4** (middle) and an authentic urine sample of a HPII patient (bottom).

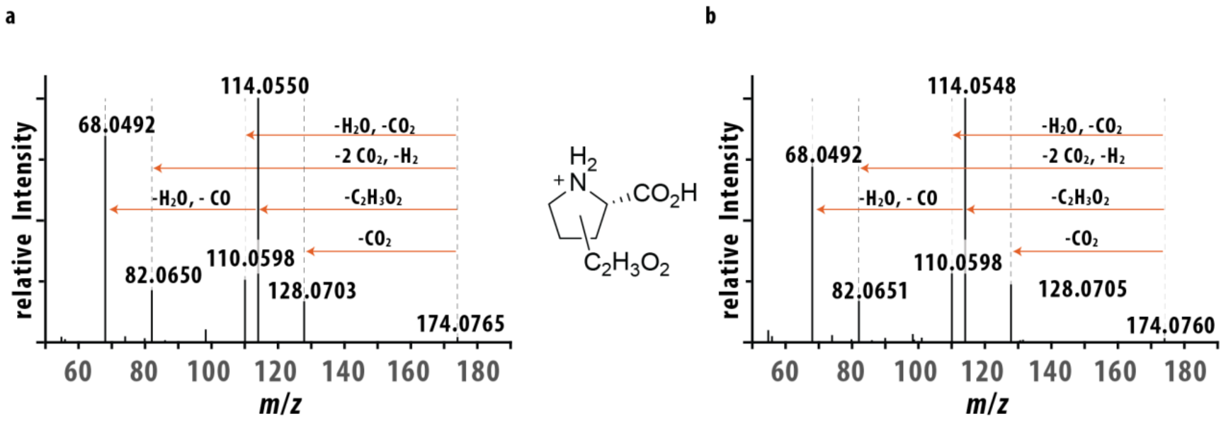


**Figure S10. CID MS/MS spectra of *m/z* 174.076. a)** Patient plasma sample (Feature C) and **b)** synthetic reference sample.

# Spectra

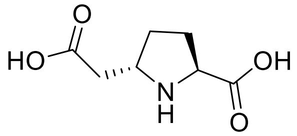


$^{1}H$ NMR (400 MHz, $D_2O$)

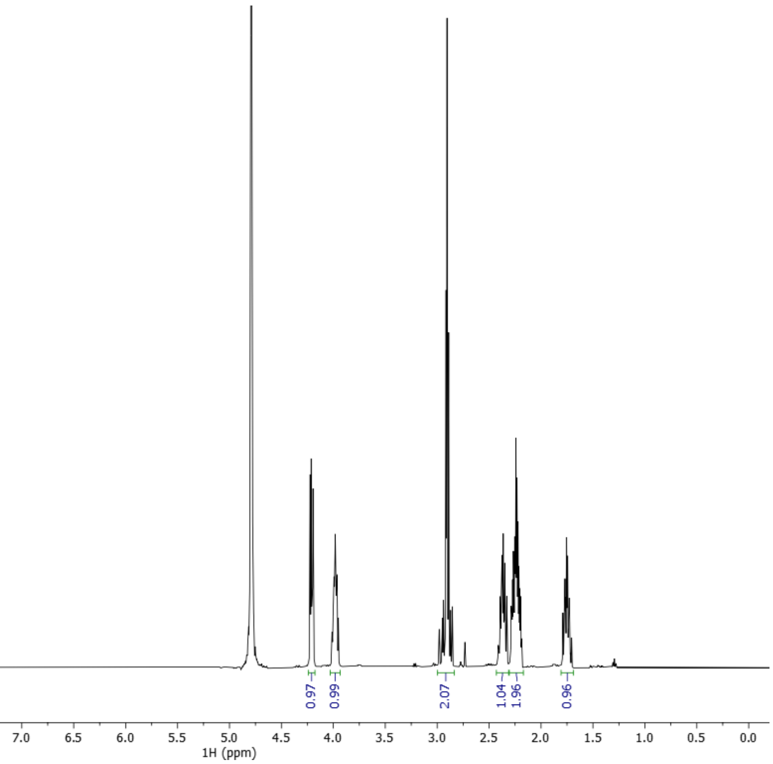


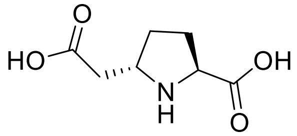


$^{13}C$ NMR (100 MHz, $D_2O$)

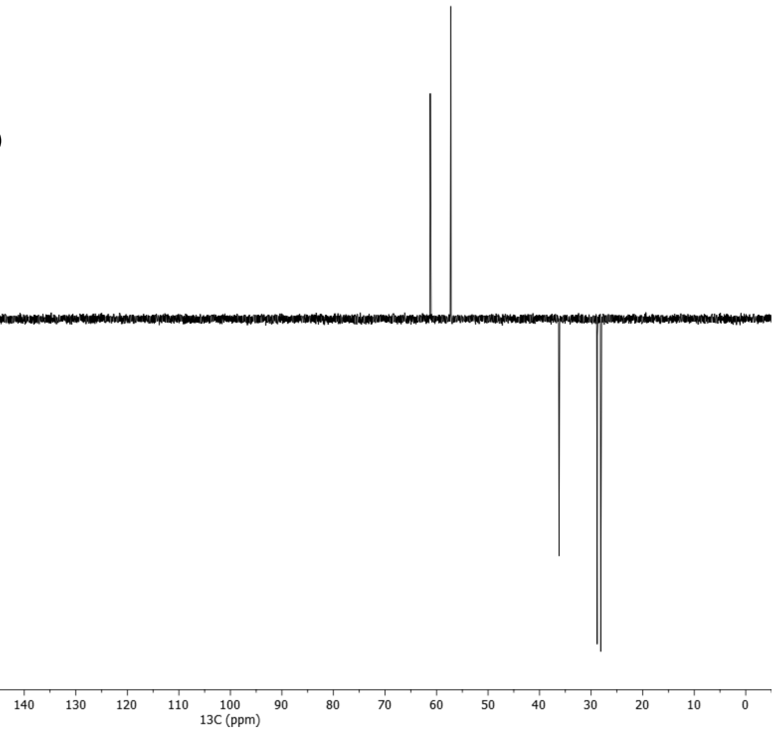

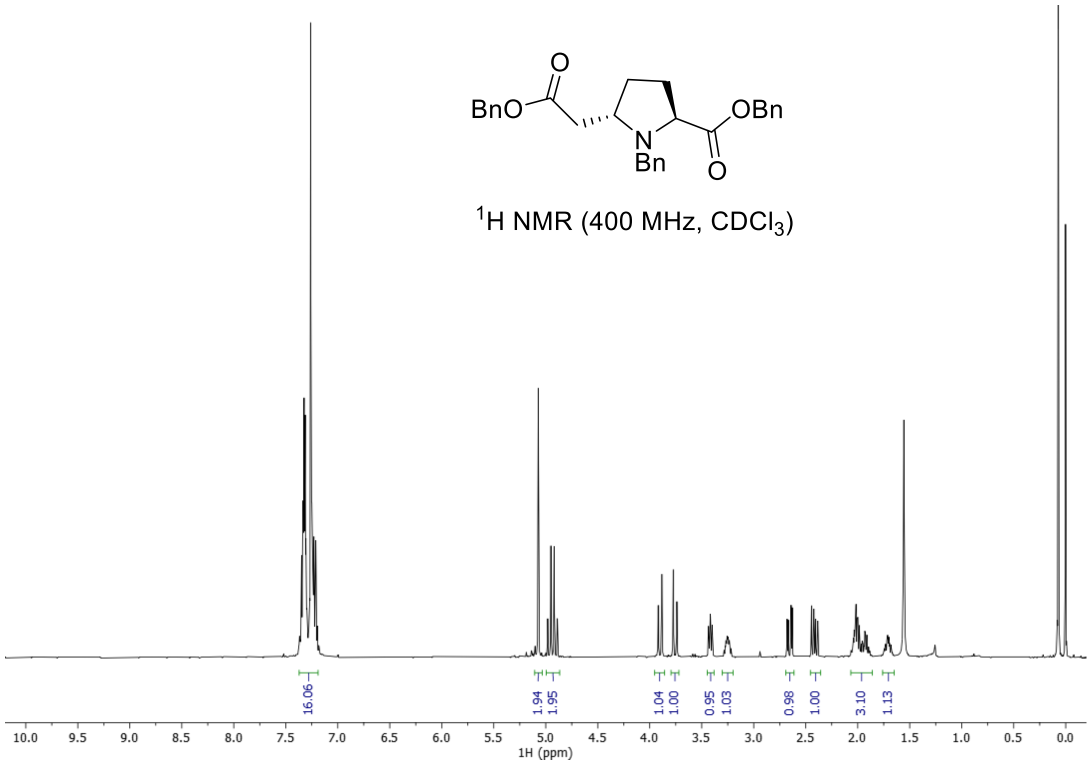
BnO
OBn
N
Bn
O
O
$^1$H NMR (400 MHz, CDCl$_3$)
16.06
1.94
1.95
1.04
1.00
0.95
1.03
0.98
1.00
3.10
1.13
10.0 9.5 9.0 8.5 8.0 7.5 7.0 6.5 6.0 5.5 5.0 4.5 4.0 3.5 3.0 2.5 2.0 1.5 1.0 0.5 0.0
1H (ppm)

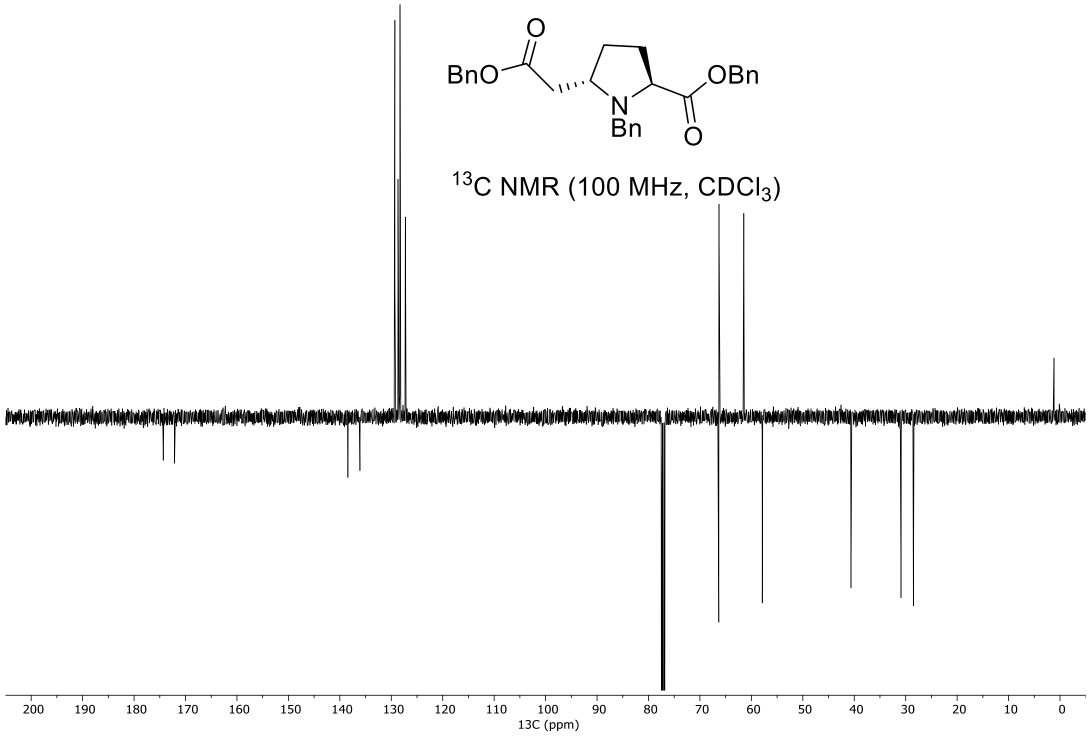
BnO
OBn
N
Bn
O
O
$^{13}$C NMR (100 MHz, CDCl$_3$)
200 190 180 170 160 150 140 130 120 110 100 90 80 70 60 50 40 30 20 10 0
13C (ppm)

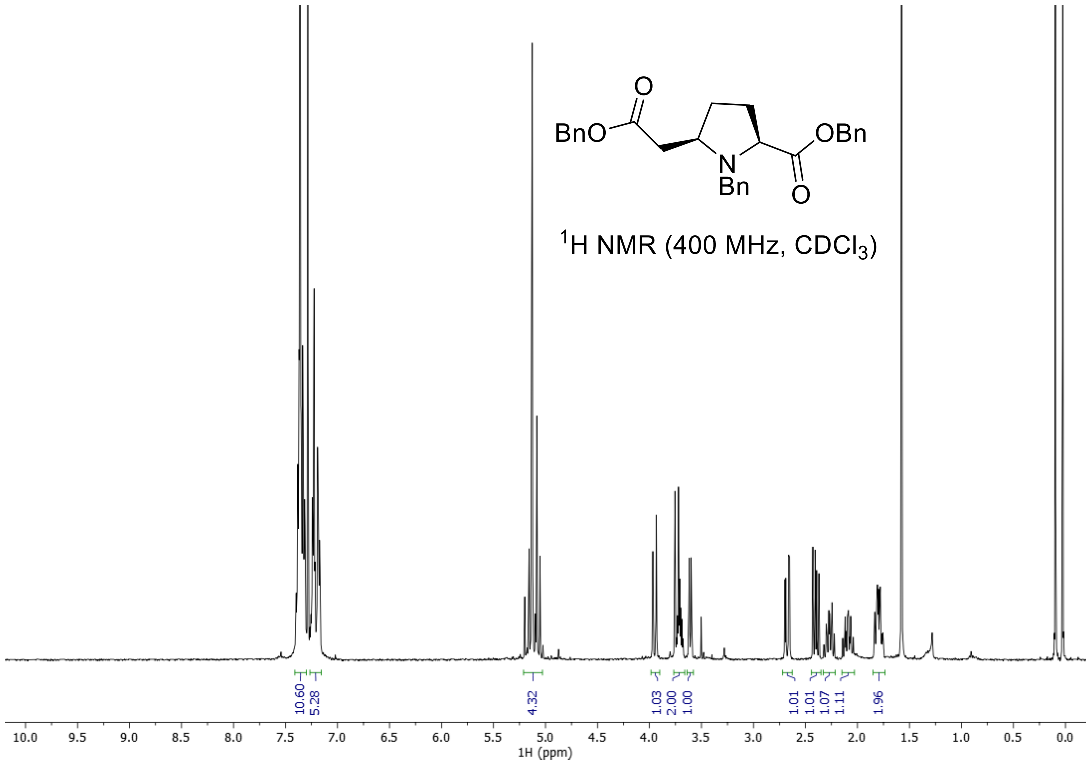
BnO
OBn
N
Bn
O
O
1H NMR (400 MHz, CDCl3)
10.60
5.28
4.32
1.03
2.00
1.00
1.01
1.01
1.07
1.11
1.96
10.0 9.5 9.0 8.5 8.0 7.5 7.0 6.5 6.0 5.5 5.0 4.5 4.0 3.5 3.0 2.5 2.0 1.5 1.0 0.5 0.0
1H (ppm)

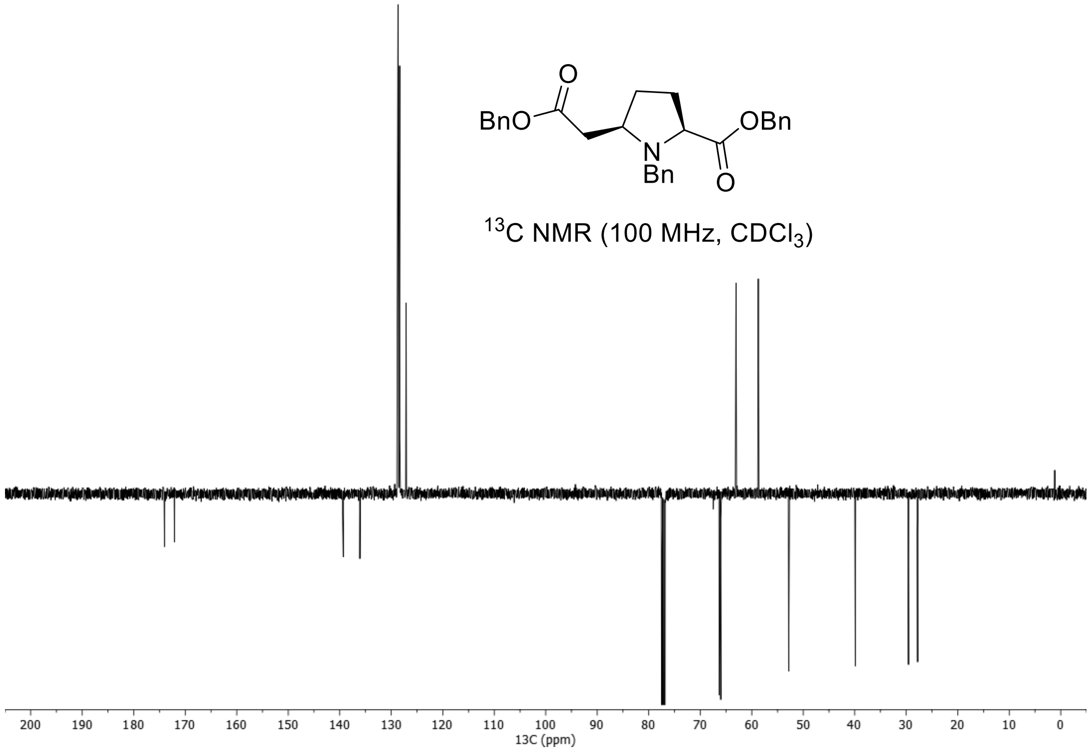
BnO
OBn
N
Bn
O
O
13C NMR (100 MHz, CDCl3)
200 190 180 170 160 150 140 130 120 110 100 90 80 70 60 50 40 30 20 10 0
13C (ppm)

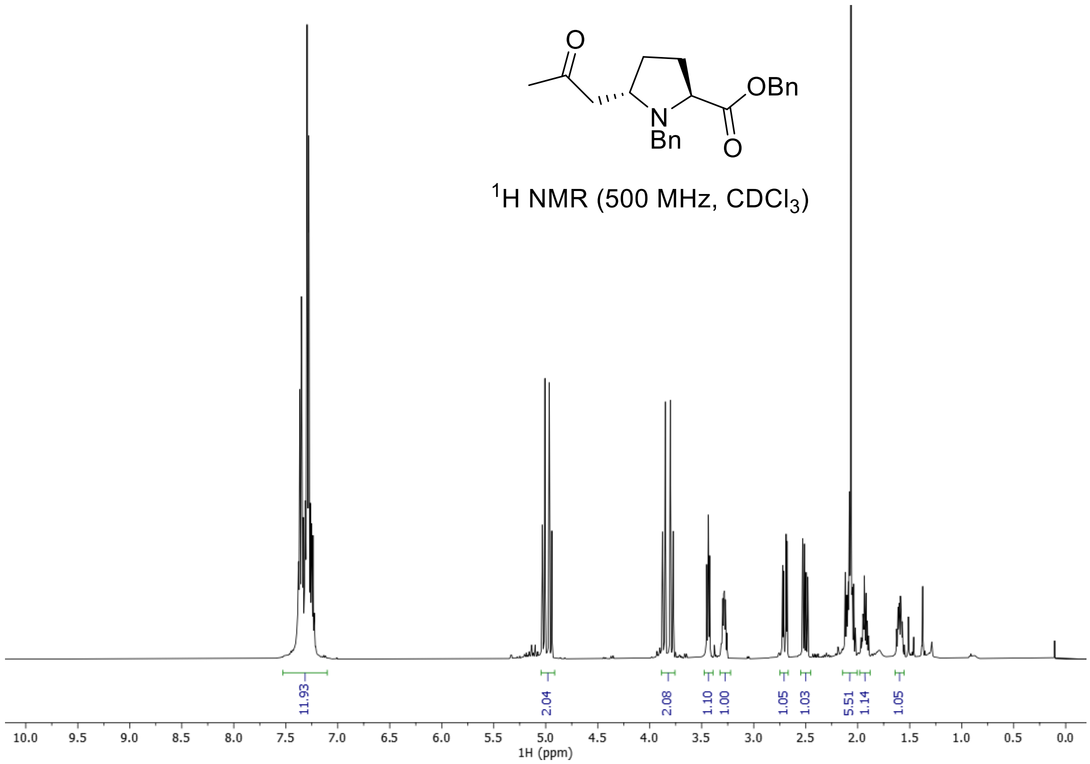
OBn
N
Bn
O
O
1H NMR (500 MHz, CDCl3)
11.93
2.04
2.08
1.10
1.00
1.05
1.03
5.51
1.14
1.05
10.0 9.5 9.0 8.5 8.0 7.5 7.0 6.5 6.0 5.5 5.0 4.5 4.0 3.5 3.0 2.5 2.0 1.5 1.0 0.5 0.0
1H (ppm)

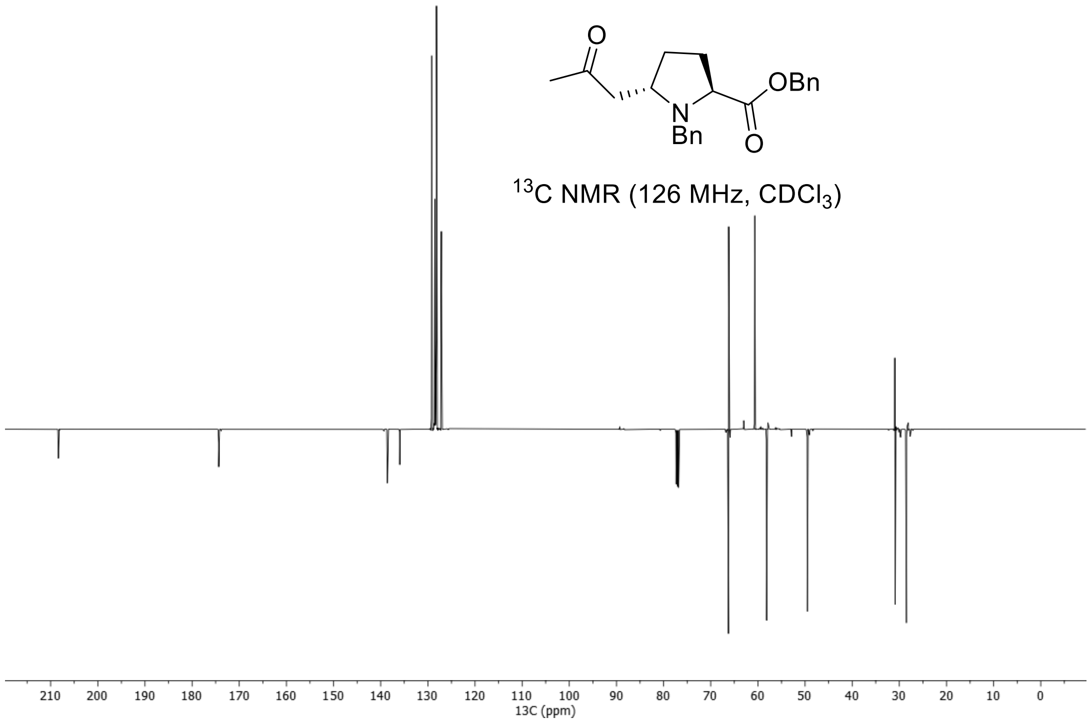
OBn
N
Bn
O
O
13C NMR (126 MHz, CDCl3)
210 200 190 180 170 160 150 140 130 120 110 100 90 80 70 60 50 40 30 20 10 0
13C (ppm)

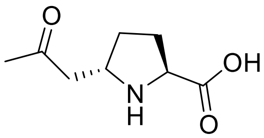


$^{1}H$ NMR (500 MHz, $D_2O$)

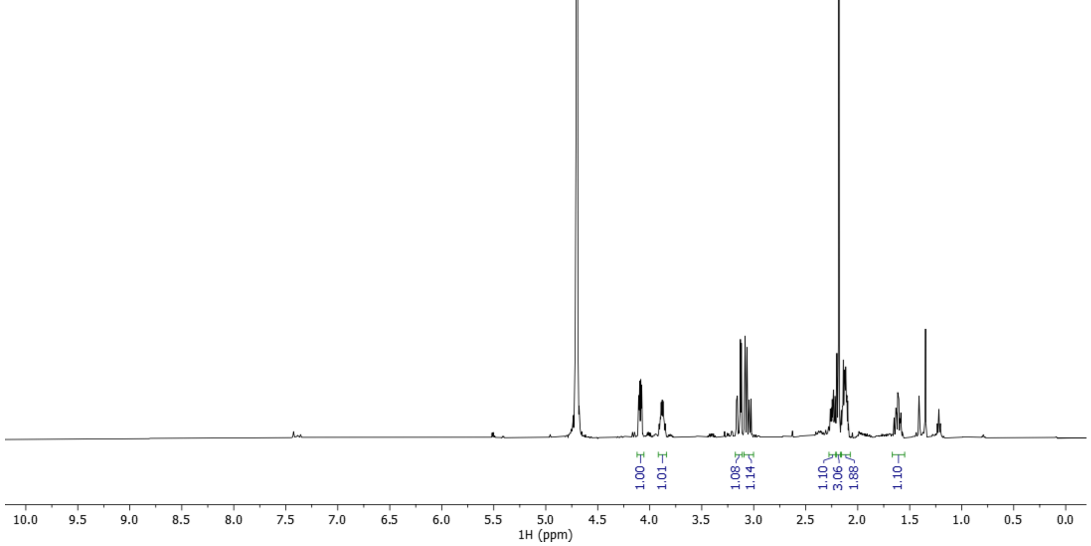


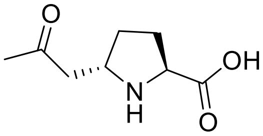


$^{13}C$ NMR (126 MHz, $D_2O$)

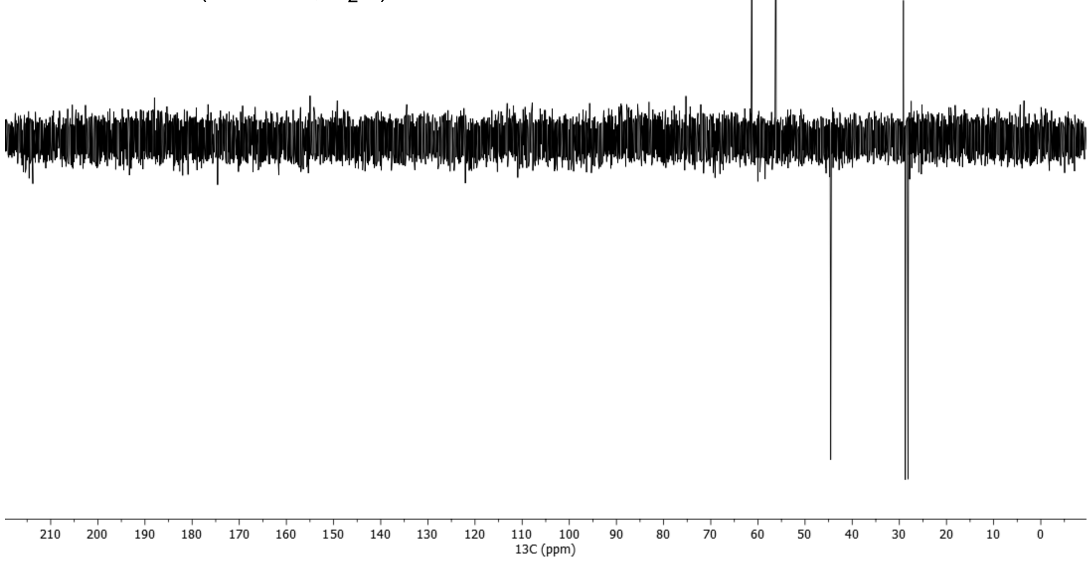

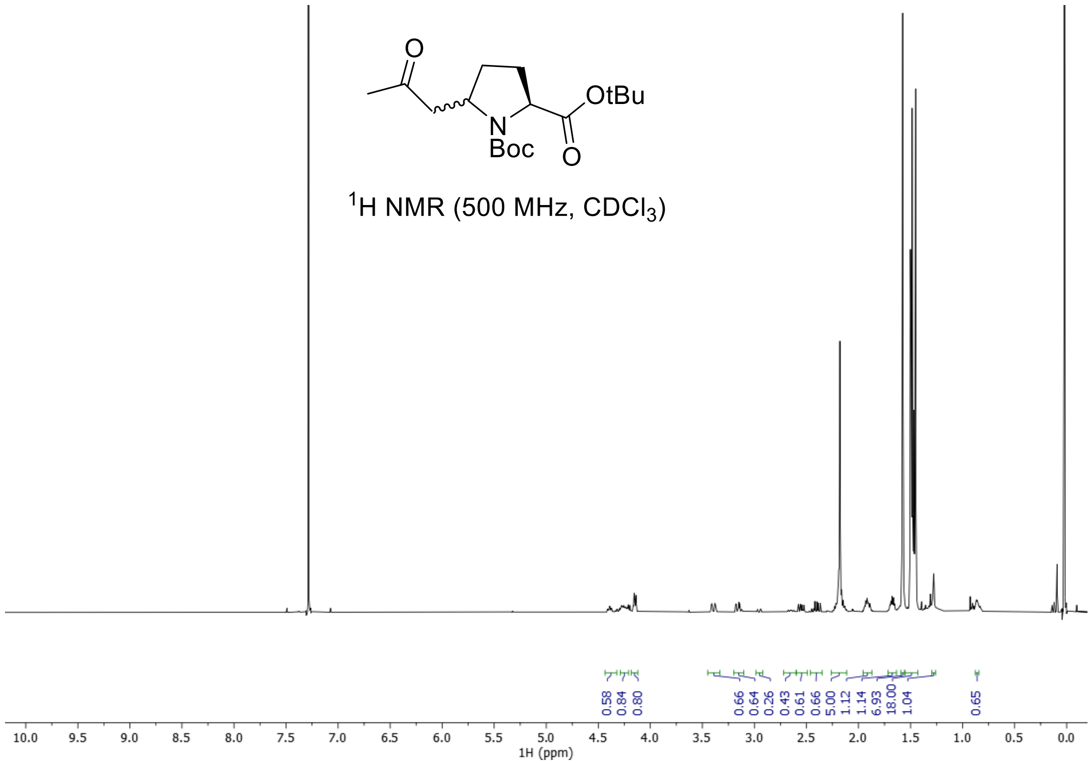
OtBu
N
Boc
O
O
1H NMR (500 MHz, CDCl3)
0.58 0.84 0.80 0.66 0.64 0.26 0.43 0.61 0.66 5.00 1.12 1.14 6.93 18.00 1.04 0.65
10.0 9.5 9.0 8.5 8.0 7.5 7.0 6.5 6.0 5.5 5.0 4.5 4.0 3.5 3.0 2.5 2.0 1.5 1.0 0.5 0.0
1H (ppm)

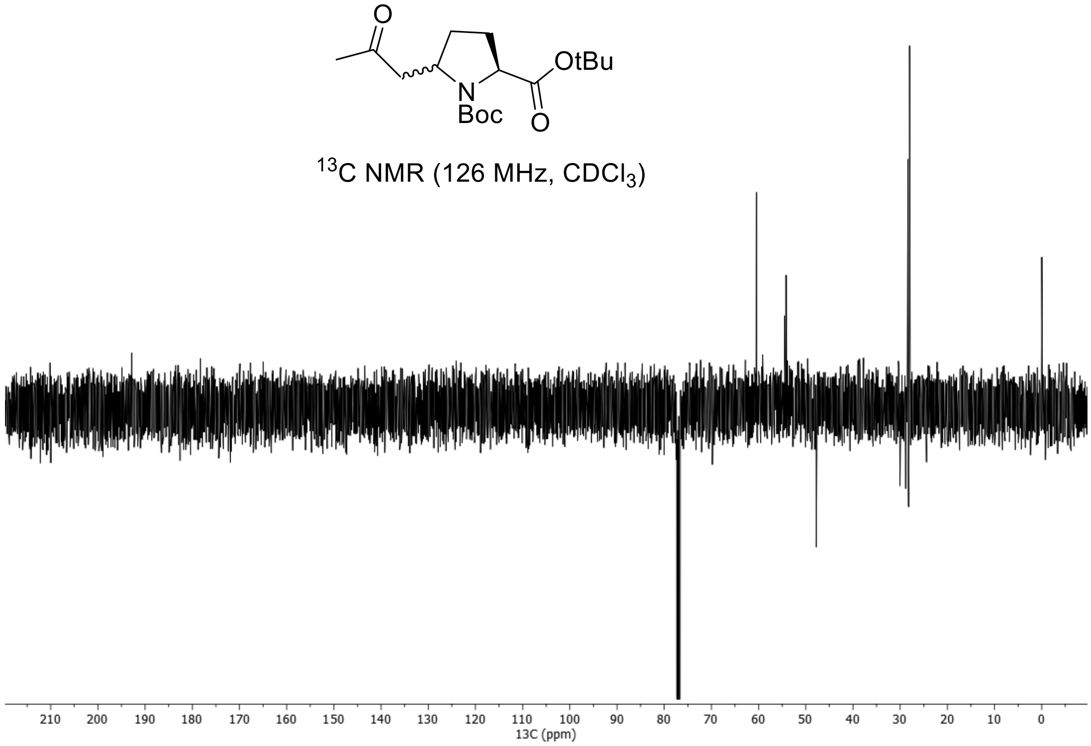
OtBu
N
Boc
O
O
13C NMR (126 MHz, CDCl3)
210 200 190 180 170 160 150 140 130 120 110 100 90 80 70 60 50 40 30 20 10 0
13C (ppm)

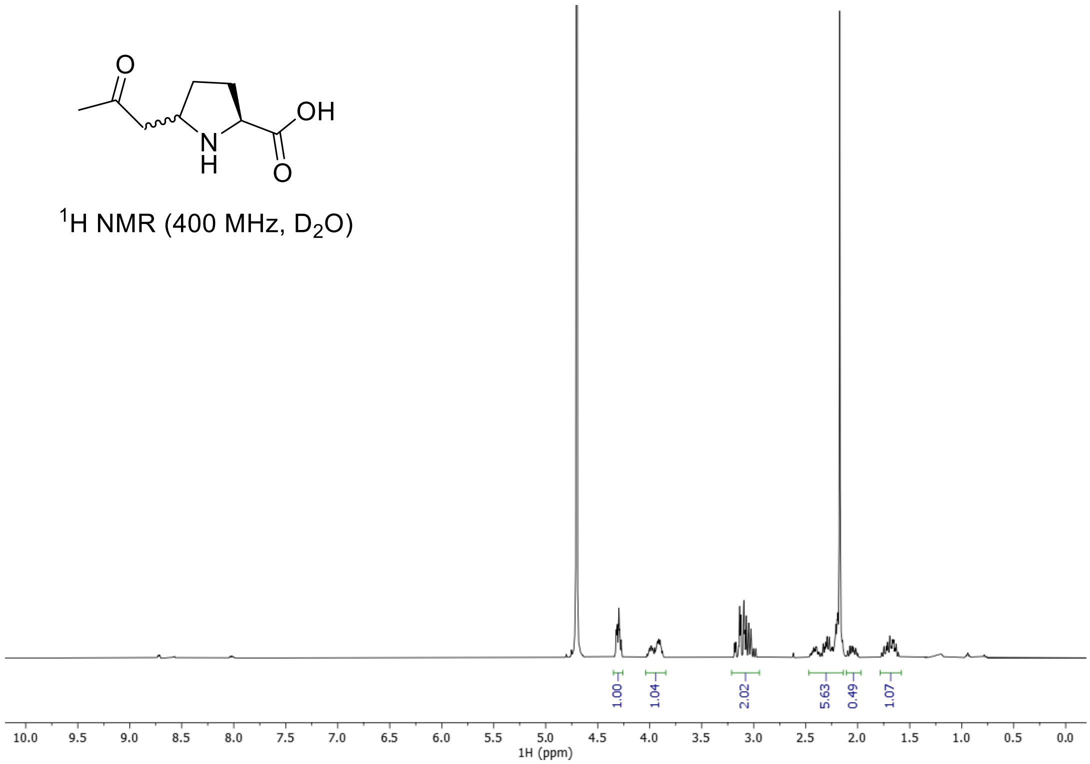
O
OH
N
H
O
1H NMR (400 MHz, D2O)
1.00
1.04
2.02
5.63
0.49
1.07
10.0 9.5 9.0 8.5 8.0 7.5 7.0 6.5 6.0 5.5 5.0 4.5 4.0 3.5 3.0 2.5 2.0 1.5 1.0 0.5 0.0
1H (ppm)

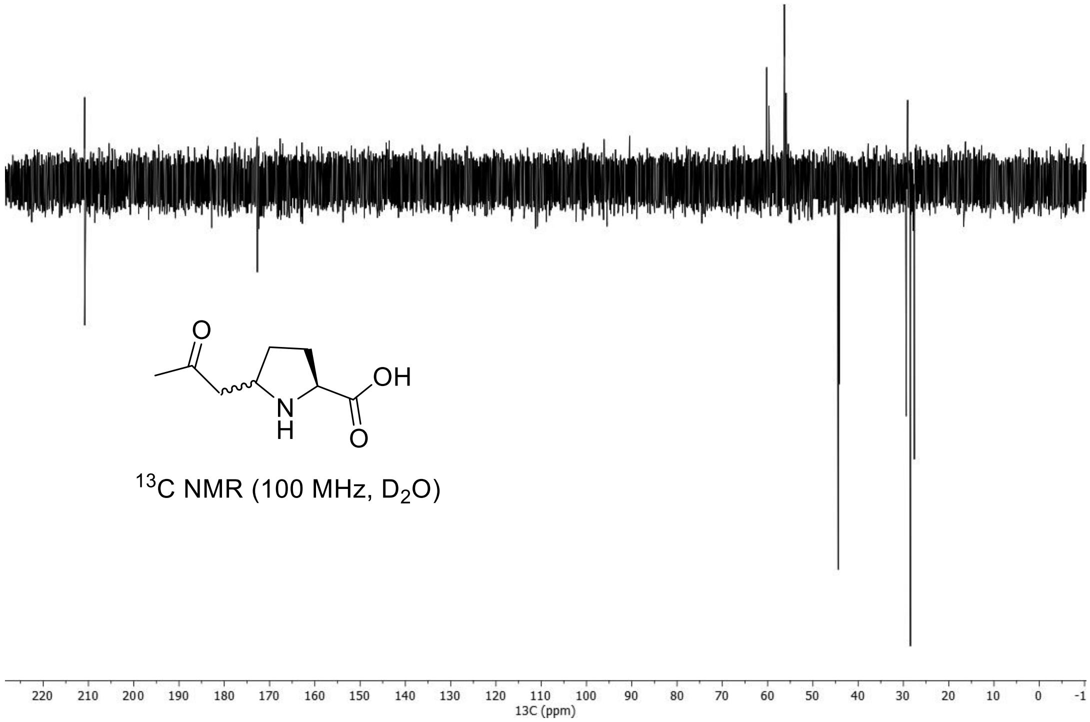
O
OH
N
H
O
13C NMR (100 MHz, D2O)
220 210 200 190 180 170 160 150 140 130 120 110 100 90 80 70 60 50 40 30 20 10 0 -1
13C (ppm)
