# Supplementary material for: Identification of Δ-1-pyrroline-5-carboxylate derived biomarkers for hyperprolinemia type II
Source: Commun Biol. 2022 Sep 21;5:997. doi: 10.1038/s42003-022-03960-2 (PMC9492674; doi:10.1038/s42003-022-03960-2)
Supplement: Supplementary file 2 — Description of Additional Supplementary Files [file 42003_2022_3960_MOESM2_ESM.pdf]

Description of Additional Supplementary Files

File name: Supplementary Data 1
Description: Data underlying the MS/MS spectra in Figure 2 and Figure S11.

File name: Supplementary Data 2
Description: Data underlying the experimental IR spectra in Figure 3 and Figure S8.

File name: Supplementary Data 3
Description: Data underlying the theoretical IR spectra and coordinates of the quantum-chemically optimized structures in Figure 3.

File name: Supplementary Data 4
Description: Data underlying the relative abundance of the m/z 172.0968 in Figure S1.

File name: Supplementary Data 5
Description: Data underlying the HPLC traces in Figures S2-7 and Figure S9.
